# Diagnostic Accuracy of Magnetic Resonance Imaging Measures of Brain Atrophy Across the Spectrum of Progressive Supranuclear Palsy and Corticobasal Degeneration

**DOI:** 10.1001/jamanetworkopen.2022.9588

**Published:** 2022-04-29

**Authors:** Ignacio Illán-Gala, Salvatore Nigro, Lawren VandeVrede, Neus Falgàs, Hilary W. Heuer, Cèlia Painous, Yaroslau Compta, Maria J. Martí, Victor Montal, Javier Pagonabarraga, Jaime Kulisevsky, Alberto Lleó, Juan Fortea, Giancarlo Logroscino, Andrea Quattrone, Aldo Quattrone, David C. Perry, Maria Luisa Gorno-Tempini, Howard J. Rosen, Lea T. Grinberg, Salvatore Spina, Renaud La Joie, Gil D. Rabinovici, Bruce L. Miller, Julio C. Rojas, William W. Seeley, Adam L. Boxer

**Affiliations:** 1 Sant Pau Memory Unit, Department of Neurology, Hospital de la Santa Creu i Sant Pau, Biomedical Research Institute Sant Pau, Universitat Autònoma de Barcelona, Barcelona, Spain; 2 Atlantic Fellow for Equity in Brain Health at the University of California, San Francisco, Department of Neurology, University of California, San Francisco; 3 Centro de Investigación en Red-Enfermedades Neurodegenerativas (CIBERNED), Madrid, Spain; 4 Center for Neurodegenerative Diseases and the Aging Brain, Department of Clinical Research in Neurology, University of Bari Aldo Moro, Pia Fondazione Cardinale G. Panico, Tricase, Lecce, Italy; 5 Institute of Nanotechnology, National Research Council, Lecce, Italy; 6 Memory and Aging Center, Department of Neurology, University of California, San Francisco; 7 Parkinson’s Disease & Movement Disorders Unit, Hospital Clínic, Instituto de Investigaciones Biomédicas August Pi i Sunyer, CIBERNED, European Reference Network for Rare Neurological Diseases, Institut de Neurociències, Universitat de Barcelona, Catalonia, Spain; 8 Movement Disorders Unit, Sant Pau Hospital and Biomedical Research Institute, Barcelona, Spain; 9 Universitat Autònoma de Barcelona, Barcelona, Spain; 10 Department of Basic Medicine, Neuroscience, and Sense Organs, University of Bari Aldo Moro, Bari, Italy; 11 Department of Medical and Surgical Sciences, Institute of Neurology, Magna Graecia University, Catanzaro, Italy; 12 Neuroimaging Research Unit, Institute of Molecular Bioimaging and Physiology, National Research Council, Catanzaro, Italy; 13 Neuroimaging Research Unit, Institute of Molecular Bioimaging and Physiology, National Research Council, Catanzaro, Italy

## Abstract

**Importance:**

The accurate diagnosis of progressive supranuclear palsy (PSP) and corticobasal degeneration (CBD) is hampered by imperfect clinical-pathological correlations.

**Objective:**

To assess and compare the diagnostic value of the magnetic resonance parkinsonism index (MRPI) and other magnetic resonance imaging–based measures of cerebral atrophy to differentiate between PSP, CBD, and other neurodegenerative diseases.

**Design, Setting, and Participants:**

This prospective diagnostic study included participants with 4-repeat tauopathies (4RT), PSP, CBD, other neurodegenerative diseases and available MRI who appeared in the University of California, San Francisco, Memory and Aging Center database. Data were collected from October 27, 1994, to September 29, 2019. Data were analyzed from March 1 to September 14, 2021.

**Main Outcomes and Measures:**

The main outcome of this study was the neuropathological diagnosis of PSP or CBD. The clinical diagnosis at the time of the MRI acquisition was noted. The imaging measures included the MRPI, cortical thickness, subcortical volumes, including the midbrain, pons, and superior cerebellar peduncle volumes. Multinomial logistic regression models (MLRM) combining different cortical and subcortical regions were defined to discriminate between PSP, CBD, and other pathologies. The areas under the receiver operating characteristic curves (AUROC) and cutoffs were calculated to differentiate between PSP, CBD, and other diseases.

**Results:**

Of the 326 included participants, 176 (54%) were male, and the mean (SD) age at MRI was 64.1 (8.0) years. The MRPI showed good diagnostic accuracy for the differentiation between PSP and all other pathologies (accuracy, 87%; AUROC, 0.90; 95% CI, 0.86-0.95) and between 4RT and other pathologies (accuracy, 80%; AUROC, 0.82; 95% CI, 0.76-0.87), but did not allow the discrimination of participants with CBD. Its diagnostic accuracy was lower in the subgroup of patients without the canonical PSP–Richardson syndrome (PSP-RS) or probable corticobasal syndrome (CBS) at MRI. MLRM combining cortical and subcortical measurements showed the highest accuracy for the differentiation between PSP and other pathologies (accuracy, 95%; AUROC, 0.98; 95% CI, 0.97-0.99), CBD and other pathologies (accuracy, 83%; AUROC, 0.86; 95% CI, 0.81-0.91), 4RT and other pathologies (accuracy, 89%; AUROC, 0.94; 95% CI, 0.92-0.97), and PSP and CBD (accuracy, 91%; AUROC, 0.95; 95% CI, 0.91-0.99), even in participants without PSP-RS or CBS at MRI.

**Conclusions and Relevance:**

In this study, the combination of widely available cortical and subcortical measures of atrophy on MRI discriminated between PSP, CBD, and other pathologies and could be used to support the diagnosis of 4RT in clinical practice.

## Key Points

**Question:**

Can widely available atrophy measures on magnetic resonance imaging (MRI) increase diagnostic accuracy of progressive supranuclear palsy (PSP) and corticobasal degeneration (CBD)?

**Findings:**

In this diagnostic study of 326 participants, different methods for quantifying cerebral atrophy on MRI at first diagnosis were applied. The combination of cortical and subcortical measures of atrophy had excellent diagnostic accuracy for the differentiation between PSP, CBD, and other pathologies, even in the subgroup of participants who did not have a movement disorder at diagnosis.

**Meaning:**

These findings suggest that structural MRI could be used to increase diagnostic certainty of underlying PSP and CBD in diverse clinically relevant scenarios.

## Teaser

This diagnostic study assesses and compares the value of the magnetic resonance parkinsonism index and other magnetic resonance imaging–based measures of cerebral atrophy to differentiate between progressive supranuclear palsy, corticobasal degeneration, and other neurodegenerative diseases.

## Introduction

Four-repeat tauopathies (4RT) are neuropathologically defined by the morphologic appearance and anatomical distribution of 4-repeat tau aggregates.^1,2^ Progressive supranuclear palsy (PSP) and corticobasal degeneration (CBD) are the 2 most common 4RT, and they represent frequent forms of late-onset frontotemporal lobar degeneration (FTLD).^3,4^ Similar to other FTLD subtypes, PSP and CBD show partially overlapping patterns of cortical neurodegeneration, mainly involving the superior frontal and perirolandic cortices.^5,6^ Unlike other causes of FTLD, 4RT are characterized by more severe subcortical neurodegeneration and variable cortical involvement.^7^

Historically, PSP and CBD have been labeled atypical parkinsonian syndromes because seminal descriptions emphasized motor features, such as akinesia, dystonia, or ocular motor abnormalities, with minimal responsiveness to levodopa therapy in most patients.^8,9^ However, over the last decades, 4RT have been associated with a wide range of phenotypes,^10,11,12,13^ including nonfluent variant primary progressive aphasia (nfvPPA), behavioral variant frontotemporal dementia (bvFTD), and an amnestic syndrome resembling Alzheimer dementia (AD).^14,15,16^

There are no effective treatments against 4RT, but increasing numbers of disease-modifying treatments are being tested.^17^ A barrier to successful 4RT trials is the lack of diagnostic biomarkers to select patients and measure treatment effects.^18,19^ Imaging biomarkers, particularly magnetic resonance imaging (MRI)–based biomarkers, have shown promise,^20^ but objective and reproducible measurements of atrophy are lacking. The recent Movement Disorders Society PSP Diagnostic Criteria update faced the challenge of insufficient evidence supporting the inclusion of neuroimaging biomarkers.^18,21^ The magnetic resonance parkinsonism index (MRPI) allows the quantification of midbrain and superior cerebellar peduncle atrophy, and provides excellent differentiation between PSP–Richardson syndrome (PSP-RS) and Parkinson disease (PD).^22^ In addition, other MRI-based measurements, such as cortical thickness and brainstem segmentations, have also shown promise for the diagnosis of 4RT,^23,24^ but their specific value for diagnosing PSP and CBD among their full spectrum of clinical presentations is unknown.

In this autopsy-confirmed study, we compared the diagnostic accuracy of antemortem MRPI and other cortical and subcortical MRI measures to differentiate among PSP, CBD, and other pathologies. We hypothesized that the combination of cortical and subcortical measures would outperform the MRPI alone and allow for improved discrimination among PSP, CBD, and other pathologies.

## Methods

### Participant Selection and Neuropathological Diagnosis

We searched the University of California, San Francisco, Memory and Aging Center (UCSF MAC) database for all patients with at least 1 MRI study (N = 4479). We excluded 4133 participants without a neuropathological diagnosis or with low-quality MRI. In patients with multiple MRI studies, we selected the first study suitable for analysis regardless of the diagnosis at MRI. This search identified a consecutive series of 326 participants with an MRI suitable for analysis and neuropathological data spanning all major neuropathological diagnoses: AD, PD, PD with Lewy body dementia, FTLD, and cerebrovascular disease. FTLD cases were further classified based on the consensus nomenclature for FTLD.^25,26,27^ Brain autopsies were performed at different brain banks following previously published methods.^16^ For the aims of this study, 3 main groups of interest were defined: PSP (68 participants), CBD (44 participants), and other pathologies (214 participants, including all other pathologies). Group details are shown in Table 1.

**Table 1.** Characteristics of the Sample

Characteristics	Participants, No. (%)			
	4RT			Other pathologies (n = 214)
	PSP (n = 68)	CBD (n = 44)	Combined (n = 112)	
Age at symptom onset, mean (SD), y	64.1 (6.98)^a^	60.0 (7)^b^	62.5 (7)^c^	57.1 (9)^b^^,^^d^
Age at MRI, mean (SD), y	69.5 (5)^a^	64.2 (6)^b^	67.4 (6)^c^	62.4 (8)^b^^,^^d^
Years of education, mean (SD)	16.2 (3)	16.1 (2)	16.2 (3)	16.2 (2)
Biological sex				
Men	32 (47.1)	19 (43.2)	51 (45.5)^c^	125 (58.4)^d^
Women	36 (52.9)	25 (56.8)	61 (54.5)^c^	89 (41.6)^d^
Diagnosis at MRI				
PSP-RS	43 (63.2)^a^	3 (6.8)^b^	46 (41.1)^c^	3 (1.4)^b^^,^^d^
CBS	11 (16.2)	12 (27.3)^c^	23 (20.5)	22 (10.3)^e^
PSP-RS or CBS	54 (79.4)^a^	15 (34.1)^f^	69 (61.6)^c^	25 (11.7)^d^^,^^g^
MMSE, mean (SD)^i^	25.5 (4.81)^c^	24.0 (6.24)	24.9 (5.44)^c^	22.6 (7.00)^b^^,^^d^
Years from MRI to death, mean (SD)	3.69 (2.01)^a^	3.25 (1.62)^b^	3.52 (1.87)^c^	4.82 (3.27)^b^^,^^d^
Primary neuropathological diagnosis				
PSP	68 (100)	0	68 (60.7)	0
CBD	0	44 (100)	44 (39.3)	0
Pick disease	0	0	0	26 (12.1)
FTLD-TDP				
Type A	0	0	0	26 (12.1)
Type B	0	0	0	34 (15.9)
Type C	0	0	0	26 (12.1)
MND-TDP	0	0	0	11 (5.1)
Other FTLD	0	0	0	32 (15.0)
AD	0	0	0	45 (21.0)
PD, LBD, MSA	0	0	0	11 (5.1)
Other	0	0	0	3 (1.4)

Abbreviations: 4RT, four-repeat tau isoform tauopathies; AD, Alzheimer disease; CBD, corticobasal disease; CBS, corticobasal syndrome; FTLD, frontotemporal lobar degeneration; LBD, Lewy body dementia; MMSE, Mini-Mental State Examination; MND, motor neuron disease; MRI, magnetic resonance image; MSA, multiple-system atrophy; PD, Parkinson disease; PSP, progressive supranuclear palsy; PSP-RS, progressive supranuclear palsy with Richardson syndrome; TDP, TAR DNA binding protein 43.

^a^ *P* < .05 compared with CBD and other pathologies.

^b^ *P* < .05 compared with PSP.

^c^ *P* < .05 compared with other pathologies.

^d^ *P* < .05 compared with combined 4RT.

^e^ *P* < .05 compared with CBD.

^f^ *P* < .05 compared with PSP and other pathologies.

^g^ *P* < .05 compared with PSP and CBD.

^i^ MMSE data was available in 307 participants (94%).

This study followed the Standards for Reporting Diagnostic Accuracy (STARD) reporting guideline. The study was approved by the UCSF institutional review board and was conducted following the Declaration of Helsinki,^28^ and written informed consent was obtained from all participants.

### Clinical Evaluation

Data were collected from October 27, 1994, to September 29, 2019. All participants included in the autopsy cohort had been clinically evaluated at the moment of MRI acquisition and received a clinical diagnosis based on patient and informant interviews, neurologic examination, and neuropsychological testing.^29^ The primary clinical syndrome at MRI was prospectively recorded, and patients were classified as PSP-RS or probable corticobasal syndrome (CBS) following previously established criteria.^12,30^ We also recorded the estimated age at symptom onset, sex, years of education, age at MRI, Mini-Mental State Examination score at the moment of MRI acquisition, and the last clinical diagnosis in participants with more than 1 visit.

### Structural MRI Acquisition and Brain Atrophy Measures

The images were acquired on 4 different MRI scans using different imaging protocols. Details on brain MRI acquisition are presented in the eMethods in the Supplement.

### Statistical Analysis

Data were analyzed from March 1 to September 14, 2021. Between-group differences in demographic variables were assessed using Mann-Whitney *U* or Kruskal-Wallis test for continuous variables and Fisher exact test for categorical data. Correlations between MRPI measures and Freesurfer segmentation–related measures of brainstem atrophy were determined with Pearson coefficients with bootstrapping-based 95% CIs.

We followed a data-driven approach to select a set of cortical and subcortical regional composites that would maximize the capacity for identifying patients with PSP and/or CBD regardless of phenotype. We defined 4 comparisons of interest: (1) PSP and other pathologies (including CBD), (2) CBD and other pathologies (including PSP); (3) 4RT (either PSP or CBD) and other pathologies; and (4) PSP and CBD (considering a clinical scenario with increased certainty of 4RT). First, we regressed out the potential confounders age, sex, total intracranial volume, and MRI scan based on an underlying fitting of regressions models. Next, Cohen *d* effect sizes were calculated using the resulting residuals for each of the 4 comparisons of interest. In a second step, we used multinomial logistic regression models (MLRM) to determine the diagnostic value of combining cortical and subcortical measures of atrophy to discriminate PSP, CBD, and other pathologies. Because of the large number of neuroimaging measures and to reduce the number of factors in MLRM, we only considered neuroimaging measures with at least a moderate effect size (as defined by absolute Cohen *d* > 0.5). We defined 2 different MLRM combining cortical and subcortical measures: one considering MRPI-derived brainstem areas (MLRM-BA) and another considering Freesurfer-derived brainstem volumes (MLRM-BV). In each MLRM, we entered age, sex, and all atrophy measures with at least a moderate effect size. We included age and sex as independent factors in each MLRM, because these variables showed statistically significant differences between the groups of interest. Finally, backward stepwise regression was used to select a unique set of cortical and subcortical regions for each MLRM. In addition, to ensure that the MLRM validated in this study could be tested in other samples, we tested additional MLRMs including raw neuroimaging measures (ie, without regressing out the potential confounders age, sex, total intracranial volume [TIV], and MRI scan). Of note, the accuracy of MLRM including unadjusted neuroimaging measures was very similar to MLRM including age-, sex-, TIV- and MRI-adjusted neuroimaging measures (eFigures 10-13 in the Supplement).

Receiver operating characteristic (ROC) curve analyses determined the diagnostic accuracy of clinical and neuroimaging measures and MLRM combining cortical and subcortical atrophy measures. To determine the diagnostic accuracy of each MLRM, we entered their estimated probabilities in ROC analyses. We calculated areas under the curve (AUROC) with 95% CIs, and we compared ROC curves with a nonparametric test that accounts for the correlation of the curves (DeLong test).^31^ Robust cutoffs maximizing the Youden index and their corresponding accuracy, sensitivity, and specificity were determined with stratified bootstrapping of 1000 samples, as implemented in the cutpointr package.^32^ The accuracy of MLRM was further validated following 5-fold cross-validation. To explore whether the diagnostic utility of neuroimaging measures and MLRM could be affected by baseline clinical characteristics, we examined ROC curves in subgroups of participants with and without a clinical diagnosis of PSP-RS or CBS.

All analyses and figures were performed using R statistical software version 4.1.1 (R Project for Statistical Computing; packages tidyverse, ggplot2, ggseg, ggstatsplot, effectsize, car, pROC, caret, nnet, cutpointr). Statistical significance for all tests was set at 5% (α = .05), all statistical tests were 2 sided, and all *P* values were corrected for multiple comparisons (Bonferroni).

## Results

### Baseline Characteristics of the Sample

Baseline characteristics of the 326 included participants are shown in Table 1. The mean (SD) age was 64.1 (8.0) years, and 176 participants (54%) were male. At the time of MRI, a diagnosis of PSP-RS was most common in the PSP group (43 of 68 [63%]), whereas a diagnosis of CBS was most common in the CBD group (12 of 44 [27%]). The 2 most common clinical diagnoses in the other pathologies group were bvFTD (97 [45%]) and CBS (22 [10%]). Overall, 43 participants with a definitive diagnosis of 4RT (48%) did not present with PSP-RS or probable CBS. Details on the predominant clinical phenotype at MRI and the last diagnosis during follow-up for each group can be found in eTable 1 in the Supplement.

### Correlations Between Brainstem Measures of Atrophy

Brainstem measurement obtained for the calculation of MRPI and their counterparts obtained with Freesurfer were highly correlated (eFigures 1-4 in the Supplement).

### Group Comparison of Measures of Brainstem Atrophy

**Figure 1.**
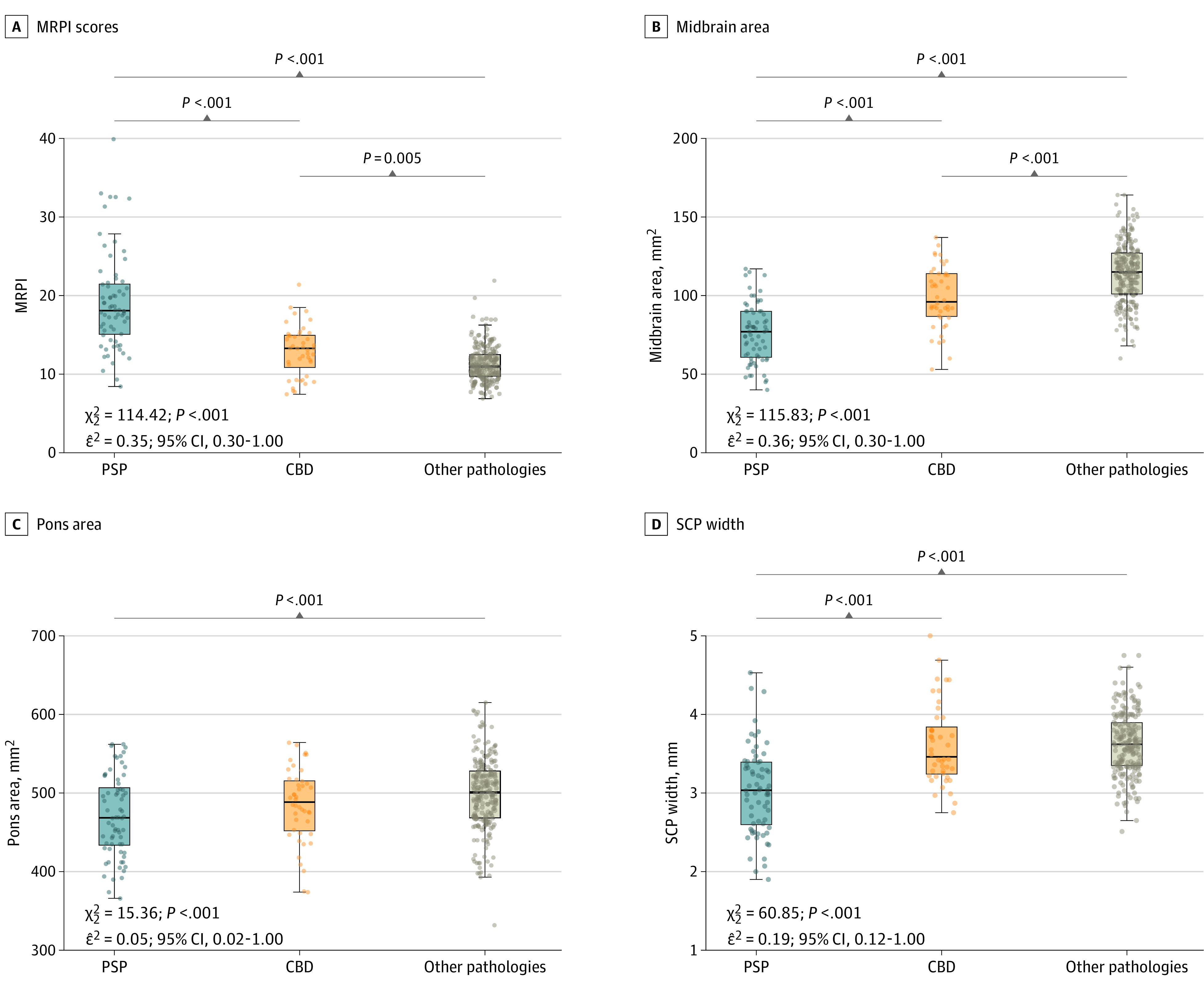
Group Comparison of Magnetic Resonance Parkinsonism Index (MRPI)–Related Measures of Brainstem Atrophy Group comparison of MRPI scores (pons area / midbrain area) × (middle cerebellar peduncle [MCP] width / superior cerebellar peduncle [SCP] width) (A), midbrain area (B), pons area (C), and SCP width. Data were analyzed using Kruskal-Wallis test followed by Wilcox post hoc analysis. Horizontal lines indicate medians; boxes, quartile 1 to quartile 3; whiskers, minimum to maximum values; and dots, individual participant values. CBD indicates corticobasal disease; and PSP, progressive supranuclear palsy.

As shown in Figure 1 and eTable 2 in the Supplement, we observed a gradient of MRPI and midsagittal midbrain area across PSP, CBD, and other pathologies. PSP had the smallest values, followed by CBD. Other pathologies had larger values than both PSP and CBD. For example, for the midsagittal midbrain area, the mean (SD) volumes were 76.1 (19.0) mm^2^ for PSP, 99.0 (20.0) mm^2^ for CBD, and 114.0 (20.0) mm^2^ for other pathologies. Similar group differences were observed when comparing the brainstem volumes obtained with Freesurfer segmentation in equivalent regions (eFigure 5 in the Supplement). Brainstem measures of atrophy were also similar within neuropathological subgroups included in the other pathologies group (eFigure 6 and eFigure 7 in the Supplement) and between participants presenting with PSP-RS or CBS and the subgroup of participants with other clinical presentations (eFigure 8 and eFigure 9 in the Supplement).

### Effect Sizes of Measures of Atrophy for the Discrimination Between Groups

**Figure 2.**
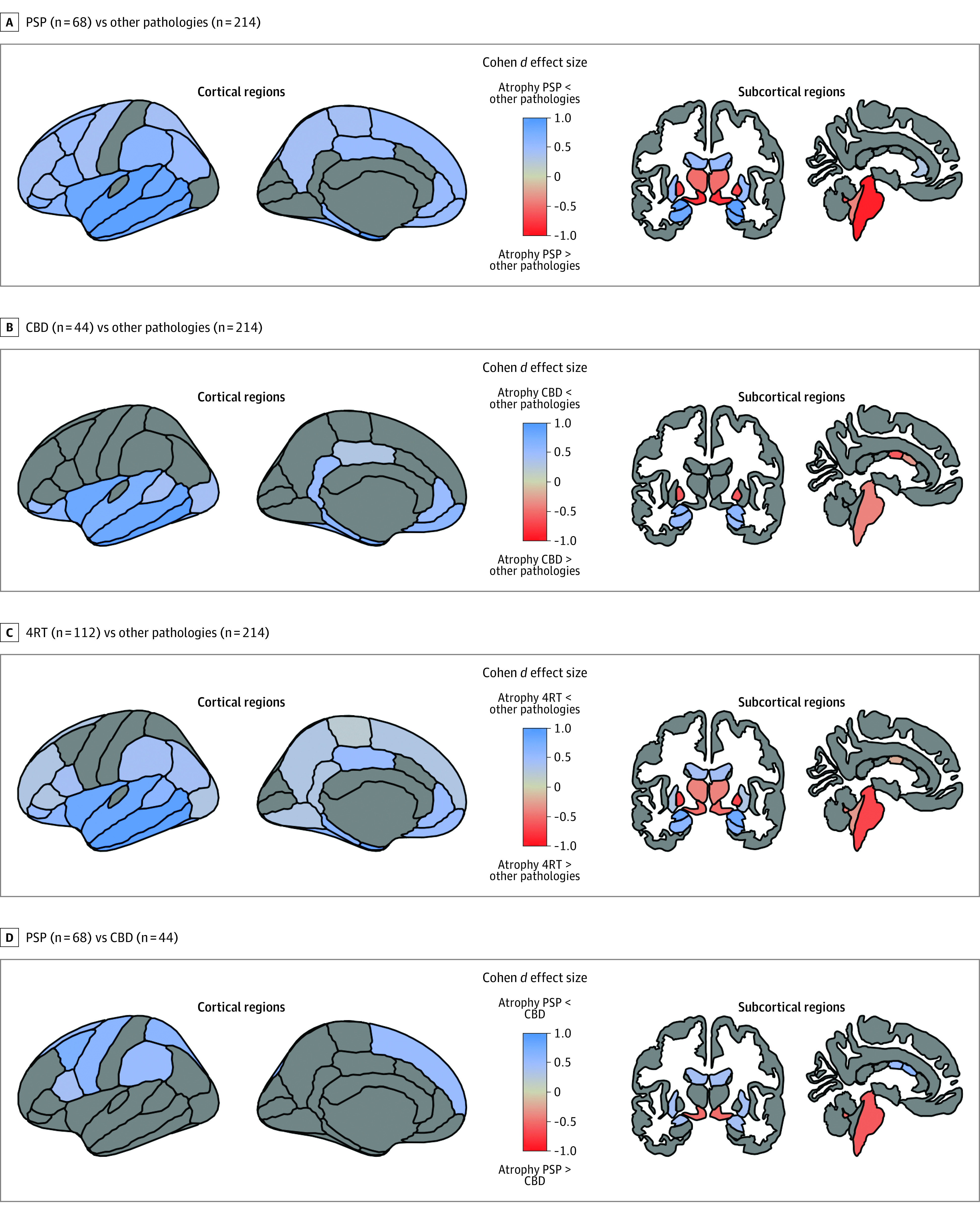
Effect Sizes of Cortical and Subcortical Measures for the Differentiation Between Groups Cortical and subcortical effect sizes for the differentiation of progressive supranuclear palsy (PSP) (n = 68) and other pathologies (n = 214) (A), corticobasal disease (CBD) (n = 44) and other pathologies (n = 214) (B), combined 4-repeat tau isoform tauopathies (4RT) (n = 112) and other pathologies (n = 214) (C), and PSP (n = 68) and CBD (n = 44) (D). Before the calculation of Cohen *d* effect sizes, potential confounders age, sex, total intracranial volume, and magnetic resonance imaging scan were regressed out in the whole sample (based on an underlying fitting of regression models). Only effect sizes for regions where statistically significant differences (*P* < .05, Bonferroni corrected) were observed are shown. The brainstem in this figure represents the whole brainstem volume. Additional information on brainstem measures can be found in Figure 1 and eTable 1 in the Supplement.

We observed a continuum of cortical atrophy between PSP and CBD with relative sparing of the cortex in PSP and intermediate levels of cortical atrophy in CBD. Participants with CBD showed relative preservation of the temporal lobe (*d* = 1.1) but similar atrophy to other pathologies in the perirolandic cortex (*d* = 0.1) (Figure 2). When compared with all other pathologies combined, both PSP and CBD showed relative preservation of the amygdala (*d* = 0.7), hippocampus (*d* = 0.6), the orbitofrontal cortex (*d* = 0.5), the insula (*d* = 0.9), and the inferior temporal cortex (*d* = 1.1), middle temporal cortex (*d* = 1.1), and superior temporal cortex (*d* = 0.8) (Figure 2). Participants in the 4RT group, however, had more atrophy in the brainstem (*d* = −0.8), ventral diencephalon (*d* = −0.8), thalamus (*d* = −0.7), and pallidum (*d* = −0.8). Despite showing partially overlapping cortical and subcortical atrophy patterns, some relevant differences between PSP and CBD groups were noted. Individuals with PSP had more atrophy than CBD in the brainstem (*d* = −0.8) and ventral diencephalon (*d* = −0.8) (Figure 2; eTable 3 in the Supplement). Conversely, CBD had more atrophy than PSP in the perirolandic cortex (*d* = −0.5), putamen (*d* = −0.2), and central and mid anterior portions of the corpus callosum (central: *d* = −0.5; mid anterior: *d* = −0.5) (Figure 2; eTable 3 in the Supplement).

### Diagnostic Accuracy of the MRPI

The MRPI showed high diagnostic accuracy for the discrimination between PSP and all other pathologies (accuracy, 87%; AUROC, 0.90; 95% CI, 0.86-0.95) but only moderate diagnostic accuracy for the discrimination between PSP and CBD (accuracy, 78%; AUROC, 0.83; 95% CI, 0.76-0.91) and 4RT vs other pathologies (accuracy, 80%; AUROC, 0.82; 95% CI, 0.76-0.87). Table 2 shows robust cutoffs that could be applied in clinically relevant scenarios. The MRPI score alone was not useful to discriminate between CBD vs all other pathologies because CBD showed intermediate levels of the MRPI score compared with PSP (highest scores) and other pathologies (lower scores).

**Table 2.** Optimal Cutoffs for MRPI and MLRM

Measure^a^	%			
	PSP vs other pathologies (including CBD)	CBD vs other pathologies (including PSP)	4RT (PSP and CBD) vs other pathologies	PSP vs CBD^b^
MRPI				
Cutoff	>14.97	NA^c^	>13.31	>16.13
Accuracy	87	NA^c^	80	78
Sensitivity	79	NA^c^	74	72
Specificity	89	NA^c^	83	88
MLRM-BA				
Cutoff	>0.30	>0.13	>0.32	>0.48
Accuracy	95	79	89	91
Sensitivity	96	96	90	90
Specificity	94	76	89	92
MLRM-BV				
Cutoff	>0.32	>0.18	>0.32	>0.48
Accuracy	92	83	86	88
Sensitivity	92	81	89	85
Specificity	92	83	85	93

Abbreviations: 4RT, 4-repeat tau isoform tauopathy; CBD, corticobasal degeneration; MRPI, magnetic resonance parkinsonism index; MLRM-BA, multinomial logistic regression model including MRPI-derived brainstem areas; MLRM-BV, multinomial logistic regression model including Freesurfer-derived brainstem volumes; NA, not assessed; PSP, progressive supranuclear palsy.

^a^ Magnetic resonance imaging–derived measurements with the highest potential to discriminate PSP, CBD, and other pathologies are shown. The MRPI and the brainstem areas considered for its calculation can be obtained online following an automated and previously validated method. To calculate estimated probabilities for MLRM-BA, MRPI-derived brainstem measures should be combined with other cortical and subcortical measures obtained following Freesurfer segmentation (Methods section and eFigure 12 in the Supplement). The calculation of estimated probabilities for MLRM-BV only requires cortical and subcortical measures obtained following Freesurfer segmentation (Methods section and eFigure 13 in the Supplement. For each biomarker and comparison of interest, the optimal cutoff and their corresponding global accuracy, sensitivity, and specificity were determined by bootstrapping 1000 samples (keeping the proportion of positives and negatives constant in every resample). In all samples, the optimal cutoff was determined with Youden index.

^b^ Cutoffs for the discrimination between PSP and CBD could be applied in samples with increased certainty of underlying PSP and CBD (ie, patients diagnosed with PSP–Richardson syndrome and probable corticobasal syndrome, after the exclusion of Alzheimer disease pathophysiology and mutations in the *GRN* gene).

^c^ Participants with CBD showed intermediate levels of the MRPI score compared with participants with PSP (highest scores) and participants with other pathologies (lower scores). Hence, the MRPI score was not useful to discriminate between participants with CBD and participants with other pathologies (including PSP).

### Comparison of Measures for the Diagnosis of Either PSP or Probable CBD

**Figure 3.**
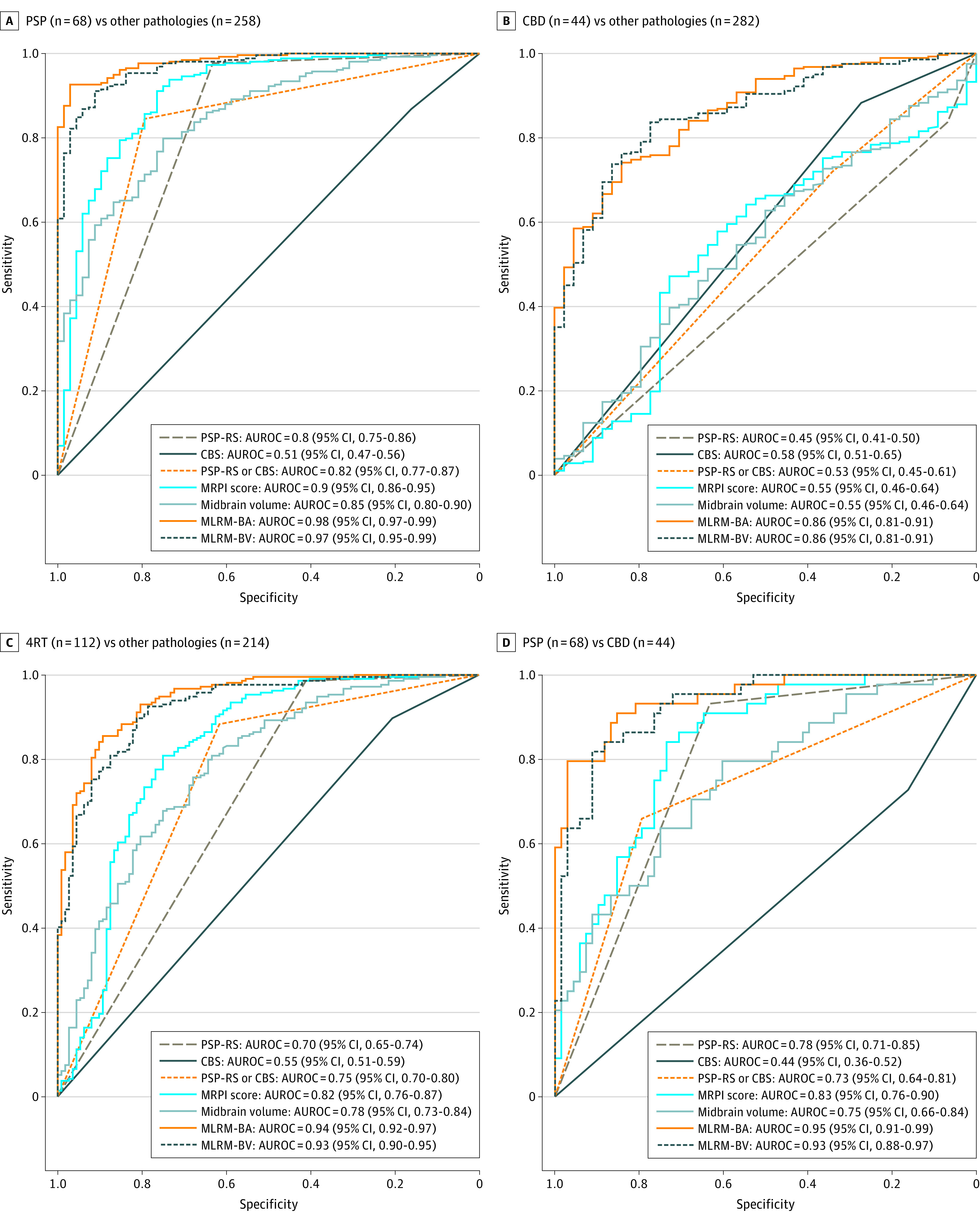
Receiver Operating Curve Analyses of Clinical Phenotypes and Relevant Measures of Cerebral Atrophy for the Discrimination Between Progressive Supranuclear Palsy (PSP), Corticobasal Disease (CBD), and Other Pathologies Receiver operating curves of clinical phenotypes, key measures of brainstem atrophy (magnetic resonance parkinsonism imaging [MRPI] score and the midbrain volume) and multinomial logistic regression model (MLRM) combining cortical and subcortical measures to discriminate among diagnoses. Details on each MLRM can be found in eFigure 10 and eFigure 11 in the Supplement. CBS indicates corticobasal syndrome; RS, Richardson syndrome.

As shown in Figure 3A and Table 2, MLRM including different cortical and subcortical regional composites yielded the highest diagnostic accuracies for the discrimination between participants with PSP and all other pathologies (MLRM-BA: accuracy, 95%; AUROC, 0.98; 95% CI, 0.97-0.99; MLRM-BV: accuracy, 92%; AUROC, 0.97; 95% CI, 0.95-0.99). Details on the characteristics of MLRM can be found in eFigures 10 to 13 in the Supplement. The AUROC for PSP vs other pathologies for MLRM were higher than for MRPI (DeLong test, MLRM-BA: *P* < .001; MLRM-BV: *P* = .01).

As shown in Figure 3B and Table 2, MLRM also showed good performance for the discrimination between CBD and the rest of the participants (MLRM-BA: accuracy, 79%; AUROC, 0.86; 95% CI, 0.81-0.91; MLRM-BV: accuracy, 83%; AUROC, 0.86; 95% CI, 0.81-0.91).

### Comparison of Measures for the Diagnosis of 4RT

When considered together, PSP and CBD were discriminated from other pathologies with high diagnostic accuracy. As shown in Figure 3C, MLRM, including cortical and subcortical atrophy measures, had excellent diagnostic accuracy for the discrimination between 4RT and other pathologies (MLRM-BA: accuracy, 89%; AUROC, 0.94; 95% CI, 0.92-0.97; MLRM-BV: accuracy, 86%; AUROC, 0.93; 95% CI, 0.90-0.95). The AUROC for 4RT vs other pathologies for the MLRM were higher than for MRPI (DeLong test, MLRM-BA: *P* = <0.001; MLRM-BV: *P* < .001).

### Comparison of Measures for the Differentiation Between PSP and CBD

As shown in Figure 3D, when restricting the analyses to the subsample of participants with either PSP or CBD, PSP-RS and CBS at MRI demonstrated a low diagnostic accuracy for the discrimination between PSP and CBD (PSP-RS: accuracy, 75%; AUROC, 0.78; 95% CI, 0.71-0.85; CBS: accuracy, 39%; AUROC, 0.44; 95% CI, 0.36-0.52). In this subsample, MLRM also showed the highest diagnostic value for the differentiation between PSP and CBD (MLRM-BA: accuracy, 91%; AUROC, 0.95; 95% CI, 0.91-0.99; MLRM-BV: accuracy, 88%; AUROC, 0.93; 95% CI, 0.88-0.97). The AUROC were also higher for MLRM vs MRPI (DeLong test, MLRM-BA: *P* = .02; MLRM-BV: *P* < .001).

### Comparison of Measures in Patients With and Without PSP-RS or CBS at MRI

We also explored whether neuroimaging measures could differentiate between underlying 4RT vs non-4RT pathologies in individuals with and without PSP-RS or CBS at MRI (eFigure 14 in the Supplement). In participants without PSP-RS or CBS, the MRPI had a lower diagnostic accuracy (accuracy, 77%; AUROC, 0.73; 95% CI, 0.63-0.83) for the detection of participants with 4RT than MLRM (MLRM-BA: accuracy, 84%; AUROC, 0.93; 95% CI, 0.90-0.97; MLRM-BV: accuracy, 84%; AUROC, 0.89; 95% CI, 0.84-0.95).

## Discussion

In this cohort study, we contrasted different MRI quantitative analyses and found that the combination of cortical and subcortical measures of atrophy had excellent diagnostic accuracy for the differentiation among participants with PSP, CBD, and other pathologies. Our findings highlight the value of automated morphometric analyses of structural MRI to support the diagnosis of PSP and CBD in diverse, clinically relevant scenarios. This may help to identify 4RT at earlier or nonmotor stages and enable accurate patient selection in clinical trials of disease-modifying therapies.

To our knowledge, this study represents the largest neuropathological validation of MRI-based biomarkers for PSP and CBD. Validating biomarkers in autopsy-proven samples is essential because clinical-pathological correlations are far from perfect. For example, in a recent clinicopathological study applying the 2017 Movement Disorders Society diagnostic criteria for PSP, as many as 32% of patients with suspected PSP did not have PSP on autopsy.^33^ The ability to determine underlying neuropathology is particularly important in CBS, for which clinical-pathological correlations are more challenging than for PSP. Previous studies have shown that the use of clinical diagnoses instead of neuropathological diagnoses may lead to a significant underestimation of the real accuracy of the biomarkers being tested and may shift diagnostic cutoffs.^34^

We observed differences in the patterns of atrophy between PSP, CBD, and other pathologies across several key subcortical structures including the midbrain, dorsal diencephalon, and pallidum. PSP showed the greatest degrees of atrophy in these subcortical structures, whereas CBD showed intermediate levels of atrophy. Individuals with other pathologies had the most prominent cortical atrophy in the frontotemporal cortices. In contrast, cortical atrophy was minimal in PSP and variable in CBD. Compared with other pathologies, both PSP and CBD had relative preservation of the temporal lobe. These observations are consistent with previous studies reporting partially overlapping patterns of neurodegeneration involving both cortical and subcortical structures^5,6,7^ in PSP and CBD and converging patterns of atrophy during follow-up.^35^

This study also provides diagnostic threshold values for the MRPI score based on autopsy confirmed cases. The MRPI score is a robust imaging biomarker developed for the detection of the typical pattern of brainstem atrophy associated with PSP.^36^ The MRPI can be obtained with a fully automated approach and has been validated in large multicenter studies.^22^ Compared with classical MRI signs, such as the so-called hummingbird and morning glory signs,^37^ the MRPI has proven to be a robust imaging biomarker for differentiating PSP-RS from PD or multiple-system atrophy.^22^ In PSP-RS and CBS, the MRPI offers good diagnostic performance for the identification of participants with underlying PSP. However, our results indicate that previously proposed diagnostic thresholds derived from clinically defined samples may identify some CBD cases presenting as PSP-RS. For example, a cut-off of 13.3 at the MRPI score was found to accurately discriminate (AUROC, 0.95) between PSP-RS from other patients presenting with parkinsonism in a large multicenter clinical cohort study.^22^ In our autopsy-proven sample, the same cutoff yielded a sensitivity of 78% and a specificity of 85% for the discrimination between PSP and other pathologies (data not shown). Our results suggest that the use of MRPI alone may not prevent the misdiagnosis of participants with CBD presenting as PSP-RS.

We also found that using only the MRPI for PSP diagnosis may miss a sizeable proportion of PSP cases, particularly in the subgroup of participants without PSP-RS or CBS. In this group, the diagnostic accuracy of the combination of cortical and subcortical measures of atrophy was superior to the diagnostic accuracy of subcortical measures alone. This result is consistent with our previous observation in participants with bvFTD who developed PSP-RS during follow-up or had PSP or CBD on autopsy.^38^ This result is also consistent with a previous study suggesting that PSP variants with prominent nonmotor signs at diagnosis may benefit from specific neuroimaging signatures, including cortical regions.^39^ Taken together, our results support the view that the MRPI can be used to increase the diagnostic certainty of either PSP or CBD in participants presenting with PSP-RS or CBS. Nonetheless, its ability to differentiate CBD from other pathologies and PSP from CBD remains limited. Of note, brainstem measurements obtained with Freesurfer (ie, midbrain or pons volumes) were highly correlated and showed similar diagnostic performance as the corresponding measurements obtained for the automated calculation of the MRPI (ie, midsagittal area of the midbrain or pons), suggesting that these 2 approaches could be used interchangeably for the diagnosis of 4RT.

The limitations of the MPRI and the other markers of subcortical atrophy together with the relative preservation of certain cortical areas in PSP and CBD justified testing alternative methods to improve the discrimination between 4RT and other pathologies. Most studies validating PSP neuroimaging biomarkers were based on clinically defined groups, focused on subcortical measurements, or only considered broad cortical regions (ie, frontal lobe or whole cerebral volume).^20^ However, this study observed a significant increase in the diagnostic performance of imaging biomarkers for the differentiation between autopsy-proven 4RT and other pathologies by combining subcortical measurements with cortical thickness in regions that are selectively affected (or spared) in PSP and CBD.

In this autopsy-confirmed cohort, we included a large sample of participants with 4RT with a wide range of clinical syndromes in addition to the canonical movement disorders associated with these diseases. Recent evidence from large multicenter studies applying modern criteria for the recognition of PSP and CBD clinical presentation suggests that focusing on classical motor presentations may either delay or miss the diagnosis of PSP or CBD.^40^ In this study, nearly half of the participants (43 [48%]) with a definitive diagnosis of 4RT did not present with PSP-RS or CBS. This observation is consistent with previous neuropathological series describing a substantial proportion of 4RT in patients presenting as bvFTD, nfvPPA, and even amnestic dementia.^7,11,15,41^ Thus, the inclusion of a substantial proportion of participants presenting with a wide range of phenotypes provides a robust validation of neuroimaging measures for the in vivo recognition of the 2 main 4RT.

### Limitations

This study has several limitations. First, we did not include detailed clinical classification according to updated PSP and CBD criteria, but we included a substantial proportion of PSP or CBD cases without a diagnosis of PSP-RS or CBS, which is still informative and avoids the selection bias of previous studies enriched with canonical motor presentations of 4RT.^42^ Second, we did not obtain cross-validation in an independent autopsy cohort because a comparable pathology-proven data set to replicate these findings is exceedingly rare. However, we performed 5-fold cross-validation to test the robustness of our results, and we provide details on logistic regression models using raw neuroimaging measures to facilitate the replication of our results in different cohorts (eFigure 12 and eFigure 13 in the Supplement). Notwithstanding, more work is needed to ensure the standardization and reproducibility of MRI-based measurements combining cortical and subcortical structures before their translation to clinical practice. Furthermore, we included a relatively small proportion of participants with alpha-synucleinopathies. Very mild cortical and brainstem changes are expected in PD or dementia with Lewy bodies, and thus, we would expect similar diagnostic accuracies in a sample enriched with alpha-synucleinopathies.

## Conclusions

In this study, the combination of widely available cortical and subcortical measures of atrophy on MRI discriminated among PSP, CBD, and other pathologies. These measures could be used to increase the recognition of 4RT as a cause of diverse neurodegenerative syndromes in clinical practice.

## Notes

Supplement. **eMethods.** Supplementary Methods
eReferences.
**eFigure 1.** Correlations Between Brainstem Measures of Atrophy **eFigure 2. **Correlation Between Brainstem, Cortical, and Subcortical Neuroimage Measures in the PSP Group **eFigure 3. **Correlation Between Brainstem, Cortical, and Subcortical Neuroimage Measures in the CBD Group **eFigure 4. **Correlation Between Brainstem, Cortical, and Subcortical Neuroimage Measures in Other Pathologies **eFigure 5. **Group Comparison of Brainstem Volumes Obtained With Freesurfer **eFigure 6. **Group Comparison of MRPI-Derived Measures of Brainstem Atrophy Within the Group of Other Pathologies **eFigure 7. **Group Comparison of Freesurfer-Derived Measures of Brainstem Atrophy Within the Group of Other Pathologies **eFigure 8. **Group Comparison of Brainstem Measures of Atrophy in the Subgroup of Participants With PSP-RS or Probable CBS at Diagnosis **eFigure 9. **Group Comparison of Brainstem Measures of Atrophy in the Subgroup of Participants Without PSP-RS or Probable CBS at Diagnosis **eFigure 10.** Multinomial Logistic Regression Model Considering Adjusted MRPI-Derived Measures of Brainstem Atrophy and Other Measures of Cortical and Subcortical Atrophy **eFigure 11. **Multinomial Logistic Regression Model Considering Adjusted Freesurfer-Derived Measures of Brainstem Atrophy and Other Measures of Cortical and Subcortical Atrophy **eFigure 12. **Multinomial Logistic Regression Models Considering Raw MRPI-Derived Measures of Brainstem Atrophy and Other Measures of Cortical and Subcortical Atrophy **eFigure 13. **Multinomial Logistic Regression Model Considering Raw Freesurfer-Derived Measures of Brainstem Atrophy and Other Measures of Cortical and Subcortical Atrophy **eFigure 14. **ROC Analyses of Relevant Measures of Cerebral Atrophy in the Subgroups of Participants With and Without a Diagnosis of PSP-RS or Probable CBS **eTable 1. **Predominant Phenotype at MRI for Each Autopsy Group **eTable 2. **Comparison of Measures of Brainstem Atrophy Across Groups **eTable 3. **Group Comparison of Neuroimaging Measures
