# Error in Article Information

**DOI:** 10.1001/jamanetworkopen.2022.17977

**Published:** 2022-05-25

In the Original Investigation titled “Diagnostic Accuracy of Magnetic Resonance Imaging Measures of Brain Atrophy Across the Spectrum of Progressive Supranuclear Palsy and Corticobasal Degeneration,”^1^ published April 29, 2022, there was an error in the Article Information section. Funding/Support should have included grants R01AG038791 and U19AG063911 from the National Institutes of Health to Dr Boxer. This article has been corrected.^1^
