# Supplementary material for: Diagnostic Accuracy of Magnetic Resonance Imaging Measures of Brain Atrophy Across the Spectrum of Progressive Supranuclear Palsy and Corticobasal Degeneration
Source: JAMA Netw Open. 2022 Apr 29;5(4):e229588. doi: 10.1001/jamanetworkopen.2022.9588 (PMC9055455; doi:10.1001/jamanetworkopen.2022.9588)
Supplement: Supplement. — eMethods. Supplementary Methods eReferences. eFigure 1. Correlations Between Brainstem Measures of Atrophy eFigure 2. Correlation Between Brainstem, Cortical, and Subcortical Neuroimage Measures in the PSP Group eFigure 3. Correlation Between Brainstem, Cortical, and Subcortical Neuroimage Measures in the CBD Group eFigure 4. Correlation Between Brainstem, Cortical, and Subcortical Neuroimage Measures in Other Pathologies eFigure 5. Group Comparison of Brainstem Volumes Obtained With Freesurfer eFigure 6. Group Comparison of MRPI-Derived Measures of Brainstem Atrophy Within the Group of Other Pathologies eFigure 7. Group Comparison of Freesurfer-Derived Measures of Brainstem Atrophy Within the Group of Other Pathologies eFigure 8. Group Comparison of Brainstem Measures of Atrophy in the Subgroup of Participants With PSP-RS or Probable CBS at Diagnosis eFigure 9. Group Comparison of Brainstem Measures of Atrophy in the Subgroup of Participants Without PSP-RS or Probable CBS at Diagnosis eFigure 10. Multinomial Logistic Regression Model Considering Adjusted MRPI-Derived Measures of Brainstem Atrophy and Other Measures of Cortical and Subcortical Atrophy eFigure 11. Multinomial Logistic Regression Model Considering Adjusted Freesurfer-Derived Measures of Brainstem Atrophy and Other Measures of Cortical and Subcortical Atrophy eFigure 12. Multinomial Logistic Regression Models Considering Raw MRPI-Derived Measures of Brainstem Atrophy and Other Measures of Cortical and Subcortical Atrophy eFigure 13. Multinomial Logistic Regression Model Considering Raw Freesurfer-Derived Measures of Brainstem Atrophy and Other Measures of Cortical and Subcortical Atrophy eFigure 14. ROC Analyses of Relevant Measures of Cerebral Atrophy in the Subgroups of Participants With and Without a Diagnosis of PSP-RS or Probable CBS eTable 1. Predominant Phenotype at MRI for Each Autopsy Group eTable 2. Comparison of Measures of Brainstem Atrophy Across Groups eTable 3. Group Comparison of Neuroi [file jamanetwopen-e229588-s001.pdf]

Supplemental Online Content

Illán-Gala I, Nigro S, VandeVrede L, et al. Diagnostic accuracy of magnetic resonance imaging measures of brain atrophy across the spectrum of progressive supranuclear palsy and corticobasal degeneration. *JAMA Netw Open.* 2022;5(4):e229588. doi:10.1001/jamanetworkopen.2022.9588

eMethods. Supplementary Methods

eReferences.

eFigure 1. Correlations Between Brainstem Measures of Atrophy

eFigure 2. Correlation Between Brainstem, Cortical, and Subcortical Neuroimage Measures in the PSP Group

eFigure 3. Correlation Between Brainstem, Cortical, and Subcortical Neuroimage Measures in the CBD Group

eFigure 4. Correlation Between Brainstem, Cortical, and Subcortical Neuroimage Measures in Other Pathologies

eFigure 5. Group Comparison of Brainstem Volumes Obtained With Freesurfer

eFigure 6. Group Comparison of MRPI-Derived Measures of Brainstem Atrophy Within the Group of Other Pathologies

eFigure 7. Group Comparison of Freesurfer-Derived Measures of Brainstem Atrophy Within the Group of Other Pathologies

eFigure 8. Group Comparison of Brainstem Measures of Atrophy in the Subgroup of Participants With PSP-RS or Probable CBS at Diagnosis

eFigure 9. Group Comparison of Brainstem Measures of Atrophy in the Subgroup of Participants Without PSP-RS or Probable CBS at Diagnosis

eFigure 10. Multinomial Logistic Regression Model Considering Adjusted MRPI-Derived Measures of Brainstem Atrophy and Other Measures of Cortical and Subcortical Atrophy

eFigure 11. Multinomial Logistic Regression Model Considering Adjusted Freesurfer-Derived Measures of Brainstem Atrophy and Other Measures of Cortical and Subcortical Atrophy

eFigure 12. Multinomial Logistic Regression Models Considering Raw MRPI-Derived Measures of Brainstem Atrophy and Other Measures of Cortical and Subcortical Atrophy

eFigure 13. Multinomial Logistic Regression Model Considering Raw Freesurfer-Derived Measures of Brainstem Atrophy and Other Measures of Cortical and Subcortical Atrophy

eFigure 14. ROC Analyses of Relevant Measures of Cerebral Atrophy in the Subgroups of Participants With and Without a Diagnosis of PSP-RS or Probable CBS

eTable 1. Predominant Phenotype at MRI for Each Autopsy Group

eTable 2. Comparison of Measures of Brainstem Atrophy Across Groups

eTable 3. Group Comparison of Neuroimaging Measures

© 2022 Illán-Gala I et al. *JAMA Network Open.*

This supplemental material has been provided by the authors to give readers additional information about their work.

© 2022 Illán-Gala I et al. *JAMA Network Open.*

eMethods. Supplementary Methods

Structural MRI acquisition and brain atrophy measures

The images were acquired on four different MRI scans using different imaging protocols. Magnetic field strength varied between 1.5 T (n=141 scans), 3.0 T (n=140 scans) and 4.0 T (n=33 scans). The MRPI was calculated with an automated algorithm that has been previously validated in a large multicenter study, including various MRI scanners.[1,2] In brief, midbrain and pons area were measured on midsagittal slice. Next, the measurement of the middle cerebellar peduncles (MCP) and superior cerebellar peduncles (SCP) widths was performed. Finally, MRPI was calculated by multiplying the pons area/midbrain area ratio by the MCP/SCP widths ratio.[2] Of note, automated brainstem segmentations were visually inspected by a human rater (S.N.), blinded to clinical diagnosis, and manually edited when needed.

Restricting the analyses to midsagittal areas and cerebellar peduncle width could be less informative than considering the whole volume of these structures because neuropathological and imaging data support that neurodegeneration in 4RT extends beyond the brainstem.[3,4] Thus, we also performed: 1) brainstem segmentation with a previously validated method implemented in Freesurfer relying on a probabilistic atlas of the brainstem and its neighboring brain structures,[5] and 2) we obtained cortical thickness, subcortical and total intracranial volumes (TIV) with the Freesurfer software (‘FreeSurfer,’ n.d., v7).[6] We further developed cortico-subcortical regional composites as described below. Mean cortical thickness was obtained for each region of interest from the 'Desikan-Killiany' cortical atlas and all cortical thickness values and subcortical volumes with one value for each hemisphere were averaged to reduce the number of measures.

© 2022 Illán-Gala I et al. *JAMA Network Open.*

Relationship between brainstem measures of atrophy

We explored the relationship between brainstem measurement obtained for the calculation of MRPI and their counterparts obtained with Freesurfer. As shown in **Supplementary Figure 1**, midsagittal areas of midbrain and pons where highly correlated with midbrain and pons volumes (r=0.72 [95% CI: 0.660 to 0.768] and r=0.78 [95% CI: 0.725 to 0.820], respectively). The SCP width showed a moderate correlation with SCP volume (r=0.45 [95% CI: 0.359 to 0.533]). We also found significant correlations between brainstem measurements, and subcortical and cortical regions in both CBD and PSP participants (**Supplementary Figures 2-4**).

Group comparison of measures of brainstem atrophy

We observed a gradient of MRPI and midsagittal midbrain area across PSP, CBD, and other pathologies (**Figure 1** and **Supplementary Table 2**). PSP group had the highest MRPI scores and the lowest midbrain area (mean [SD], 19.2 [6.2], and 76.1 [18.6], respectively). In contrast, the other pathologies group had the lowest MRPI scores and the highest midbrain area (mean [SD], 11.3 [2.3], and 114.1 [19.6], respectively). CBD displayed intermediate MRPI scores and midbrain area (mean [SD], 13.0 [3.2] and 99.0 [19.7], respectively). SCP width was reduced in PSP compared to other pathologies and CBD. Similar group differences were observed when comparing the brainstem volumes obtained with Freesurfer segmentation in equivalent regions **(Supplementary Figure 5)**. Brainstem measures of atrophy were similar within neuropathological subgroups included in the "other pathologies" group (**Supplementary Figures 6 and 7**).

We also explored if group differences in measures of brainstem atrophy differed between the subgroup of participants presenting with PSP-RS or CBS and the subgroup of participants with other clinical presentations. Within the participants presenting with PSP-RS or CBS, those with

© 2022 Illán-Gala I et al. *JAMA Network Open.*

PSP pathology also had higher MRPI, lower midbrain area/volume, and lower SCP width/volume than those with other pathologies (**Supplementary Figure 8**). Within the subgroup of participants presenting with other phenotypes, those with PSP pathology also had higher MRPI and lower midbrain area/volume but similar pons area/volume and SCP width/volume than those with other pathologies (**Supplementary Figure 9**).

eReferences

1. Nigro S, Antonini A, Vaillancourt DE, et al. Automated MRI Classification in Progressive Supranuclear Palsy: A Large International Cohort Study. *Mov Disord*. 2020;35(6):976-983. doi:10.1002/mds.28007

2. Nigro S, Arabia G, Antonini A, et al. Magnetic Resonance Parkinsonism Index: diagnostic accuracy of a fully automated algorithm in comparison with the manual measurement in a large Italian multicentre study in patients with progressive supranuclear palsy. *Eur Radiol*. 2017;27(6):2665-2675. doi:10.1007/s00330-016-4622-x

3. Cordato NJ, Halliday GM, Harding AJ, Hely MA, Morris JG. Regional brain atrophy in progressive supranuclear palsy and Lewy body disease. *Ann Neurol*. 2000;47(6):718-728.

4. Dutt S, Binney RJ, Heuer HW, et al. Progression of brain atrophy in PSP and CBS over 6 months and 1 year. *Neurology*. 2016;87(19):2016-2025. doi:10.1212/WNL.0000000000003305

5. Iglesias JE, Van Leemput K, Bhatt P, et al. Bayesian segmentation of brainstem structures in MRI. *NeuroImage*. 2015;113:184-195. doi:10.1016/j.neuroimage.2015.02.065

6. Fischl B, Dale AM. Measuring the thickness of the human cerebral cortex from magnetic resonance images. *Proc Natl Acad Sci*. 2000;97(20):11050-11055.

© 2022 Illán-Gala I et al. *JAMA Network Open.*

eFigure 1. Correlations Between Brainstem Measures of Atrophy

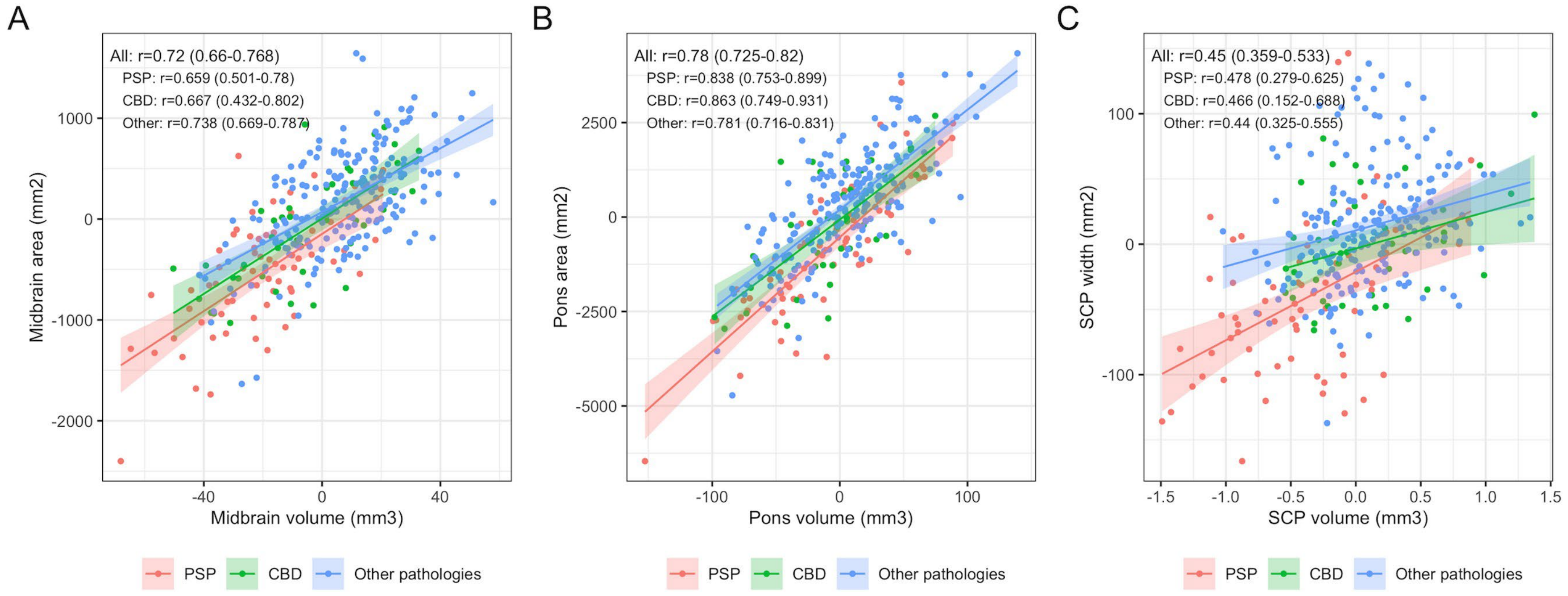

Partial correlations between brainstem measures of atrophy accounting for the effect of age, sex, TIV and MRI scan are shown. Bootstrapping-based 95% confidence intervals are displayed between parentheses.

Abbreviations: CBD=corticobasal disease; PSP=progressive supranuclear palsy; ns=non-significant; TIV=total intracranial volume; MRPI=magnetic resonance parkinsonism index; SCP=superior cerebellar peduncle.

© 2022 Illán-Gala I et al. *JAMA Network Open.*

eFigure 2. Correlation Between Brainstem, Cortical, and Subcortical Neuroimage Measures in the PSP Group (n=68)

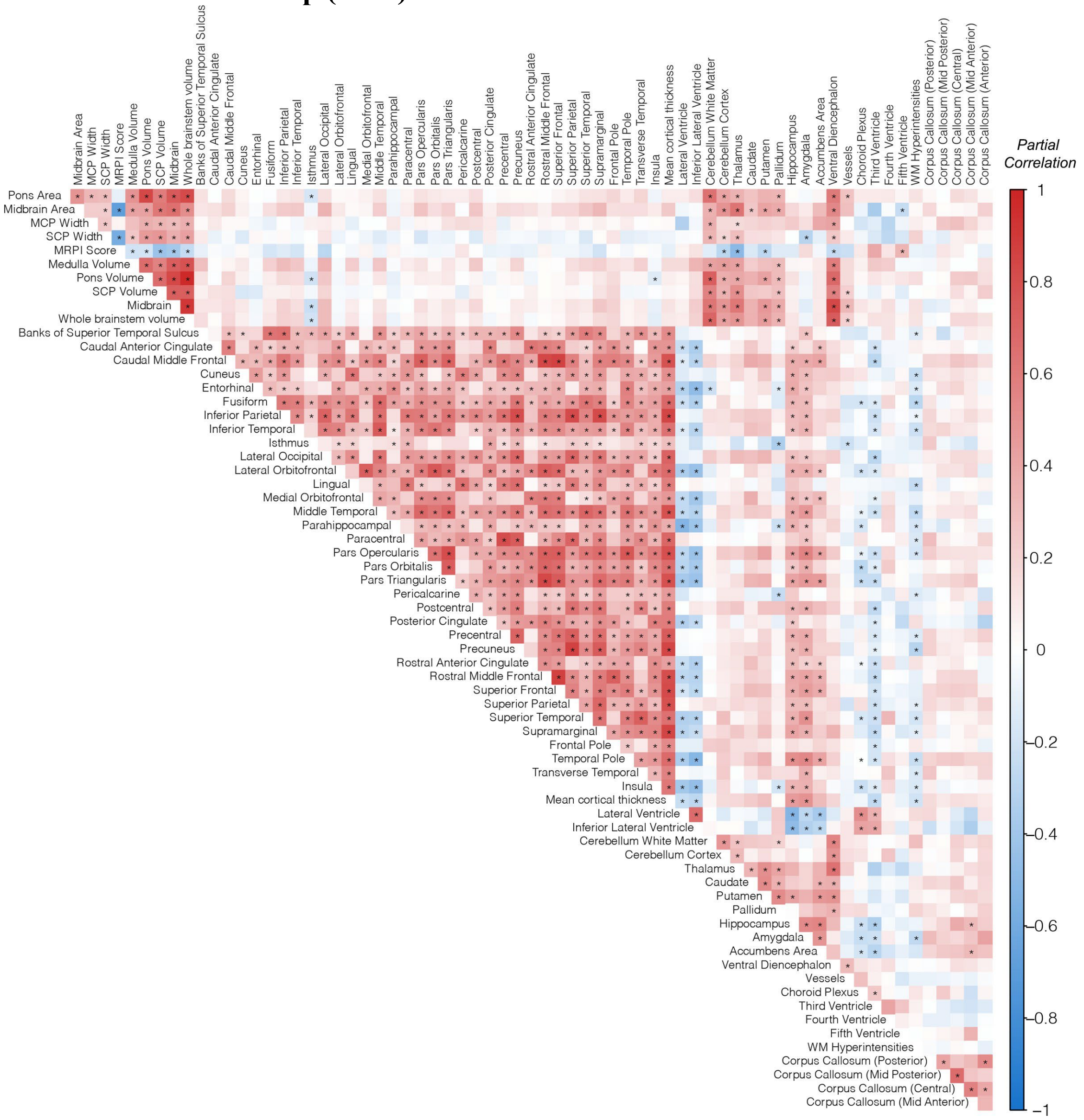

Partial correlations between brainstem measures of atrophy accounting for the effect of age, sex, TIV and MRI scan are shown. Asterisks represent statistically significant correlations (*P*<0.05, Bonferroni-corrected).

Abbreviations: PSP=progressive supranuclear palsy; MCP=middle cerebellar peduncle; MRPI=magnetic resonance parkinsonism index; SCP=superior cerebellar peduncle; WM=white matter

© 2022 Illán-Gala I et al. *JAMA Network Open.*

eFigure 3. Correlation Between Brainstem, Cortical, and Subcortical Neuroimage Measures in the CBD Group (n=44)

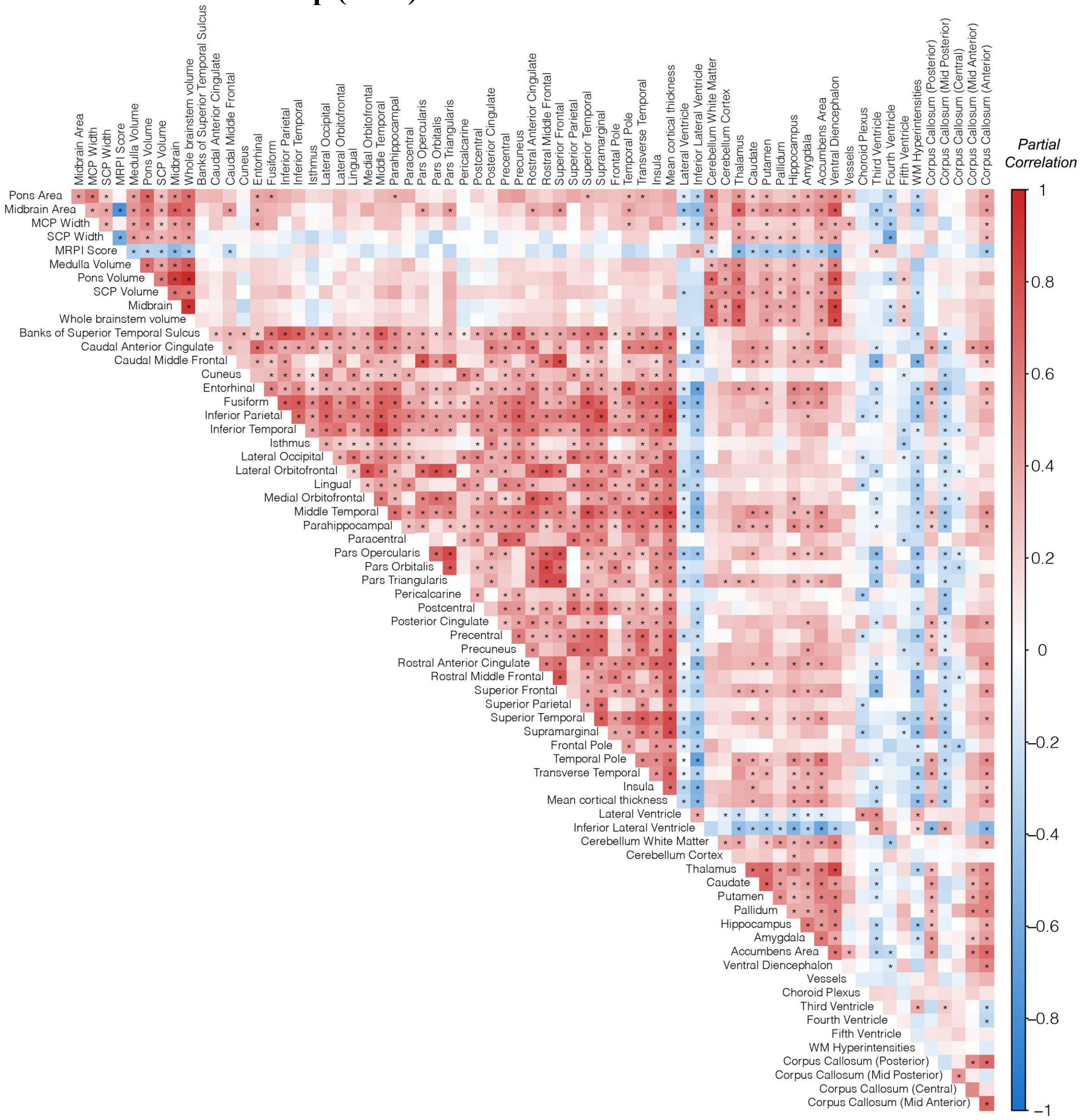

Partial correlations between brainstem measures of atrophy accounting for the effect of age, sex, TIV and MRI scan are shown. Asterisks represent statistically significant correlations ($P<0.05$, Bonferroni-corrected).

Abbreviations: CBD=corticobasal disease; MCP=middle cerebellar peduncle; MRPI=magnetic resonance parkinsonism index; SCP=superior cerebellar peduncle; WM=white matter

© 2022 Illán-Gala I et al. *JAMA Network Open.*

eFigure 4. Correlation Between Brainstem, Cortical, and Subcortical Neuroimage Measures in Other Pathologies (n=214)

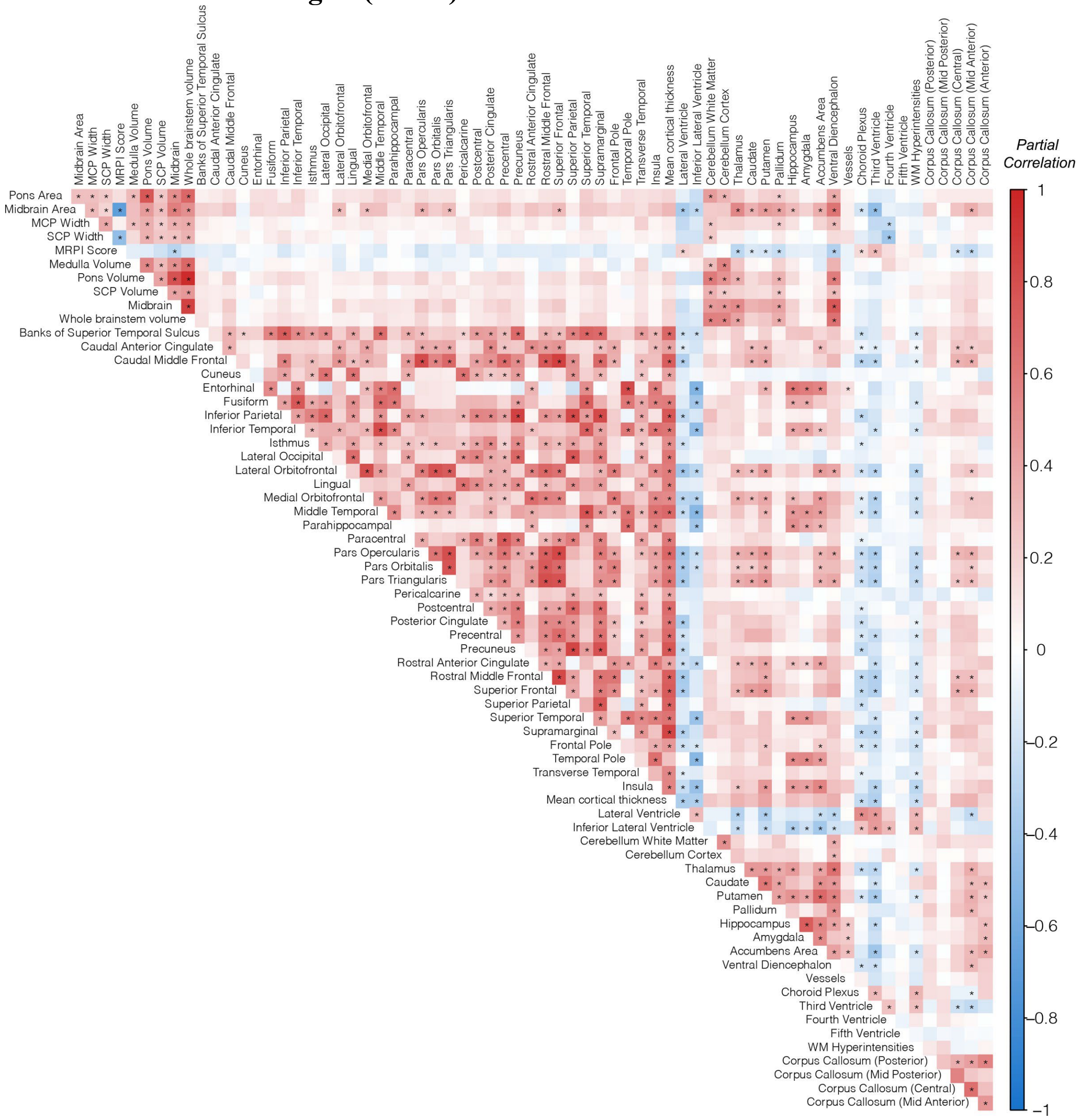

Partial correlations between brainstem measures of atrophy accounting for the effect of age, sex, TIV and MRI scan are shown. Asterisks represent statistically significant correlations (*P*<0.05, Bonferroni-corrected).

Abbreviations: MCP=middle cerebellar peduncle; MRPI=magnetic resonance parkinsonism index; SCP=superior cerebellar peduncle; WM=white matter

© 2022 Illán-Gala I et al. *JAMA Network Open.*

eFigure 5. Group Comparison of Brainstem Volumes Obtained With Freesurfer

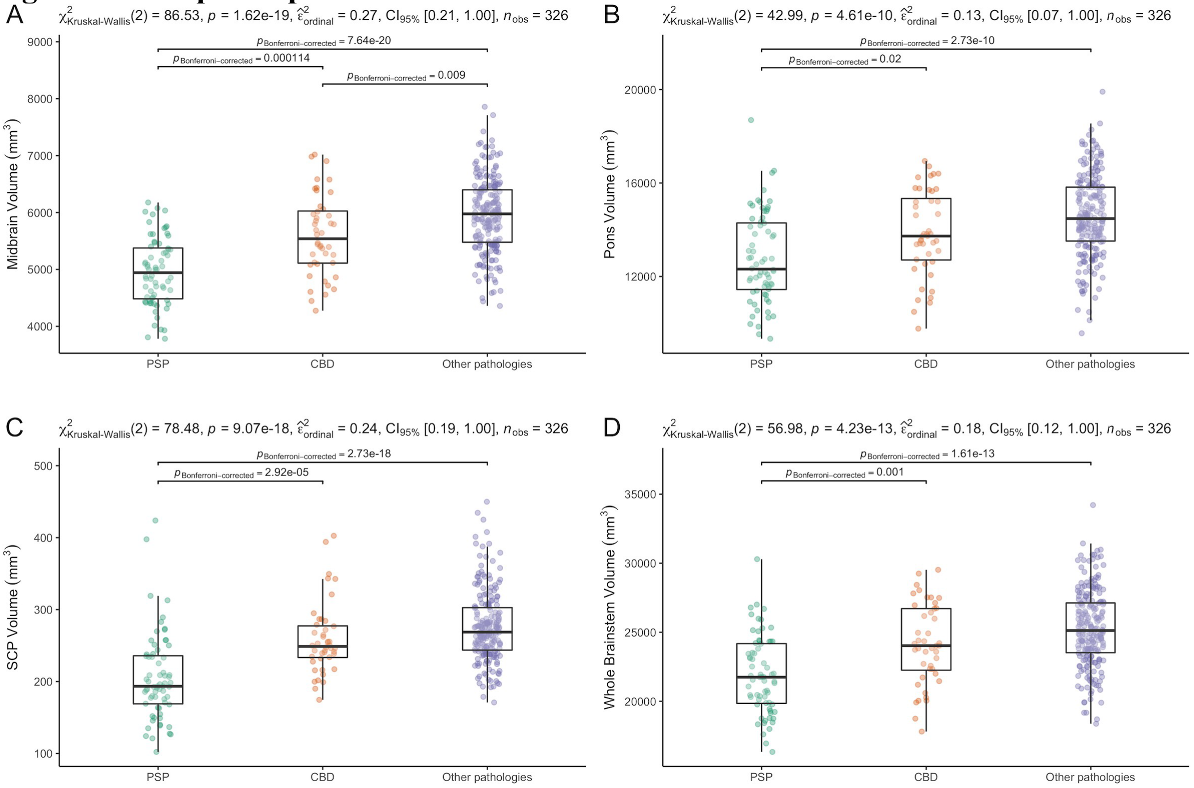

Group comparison of **A)** midbrain volume, **B)** pons volume, **C)** SCP volume, **D)** whole brainstem volume calculated with Freesurfer. Data were analyzed using Kruskal Wallis test followed by Wilcox post-hoc analysis after accounting for the effect of age, sex, TIV and MRI scan. Horizontal lines indicate medians; boxes, quartile 1 to quartile 3; whiskers, minimum to maximum values; and dots, individual participant values.

Abbreviations: CBD=corticobasal disease; PSP=progressive supranuclear palsy; ns=non-significant; SCP=superior cerebellar peduncle.

© 2022 Illán-Gala I et al. *JAMA Network Open.*

eFigure 6. Group Comparison of MRPI-Derived Measures of Brainstem Atrophy Within the Group of Other Pathologies

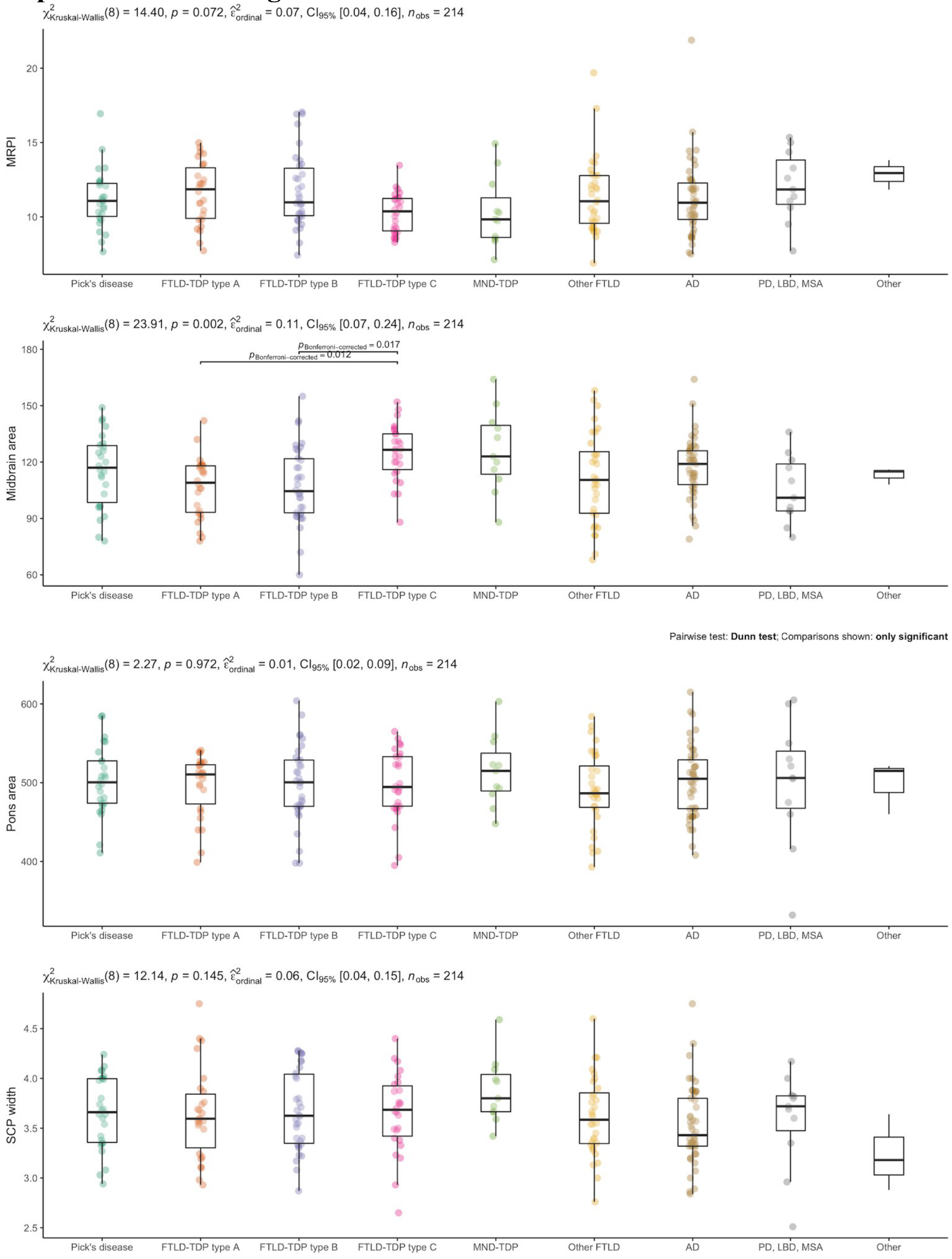

Horizontal lines indicate medians; boxes, quartile 1 to quartile 3; whiskers, minimum to maximum values; and dots, individual participant values.

Abbreviations: CBD=corticobasal disease; PSP=progressive supranuclear palsy; ns=non-significant; MCP=middle cerebellar peduncle; MRPI=magnetic resonance parkinsonism index; SCP=superior cerebellar peduncle.

© 2022 Illán-Gala I et al. *JAMA Network Open.*

eFigure 7. Group Comparison of Freesurfer-Derived Measures of Brainstem Atrophy Within the Group of Other Pathologies

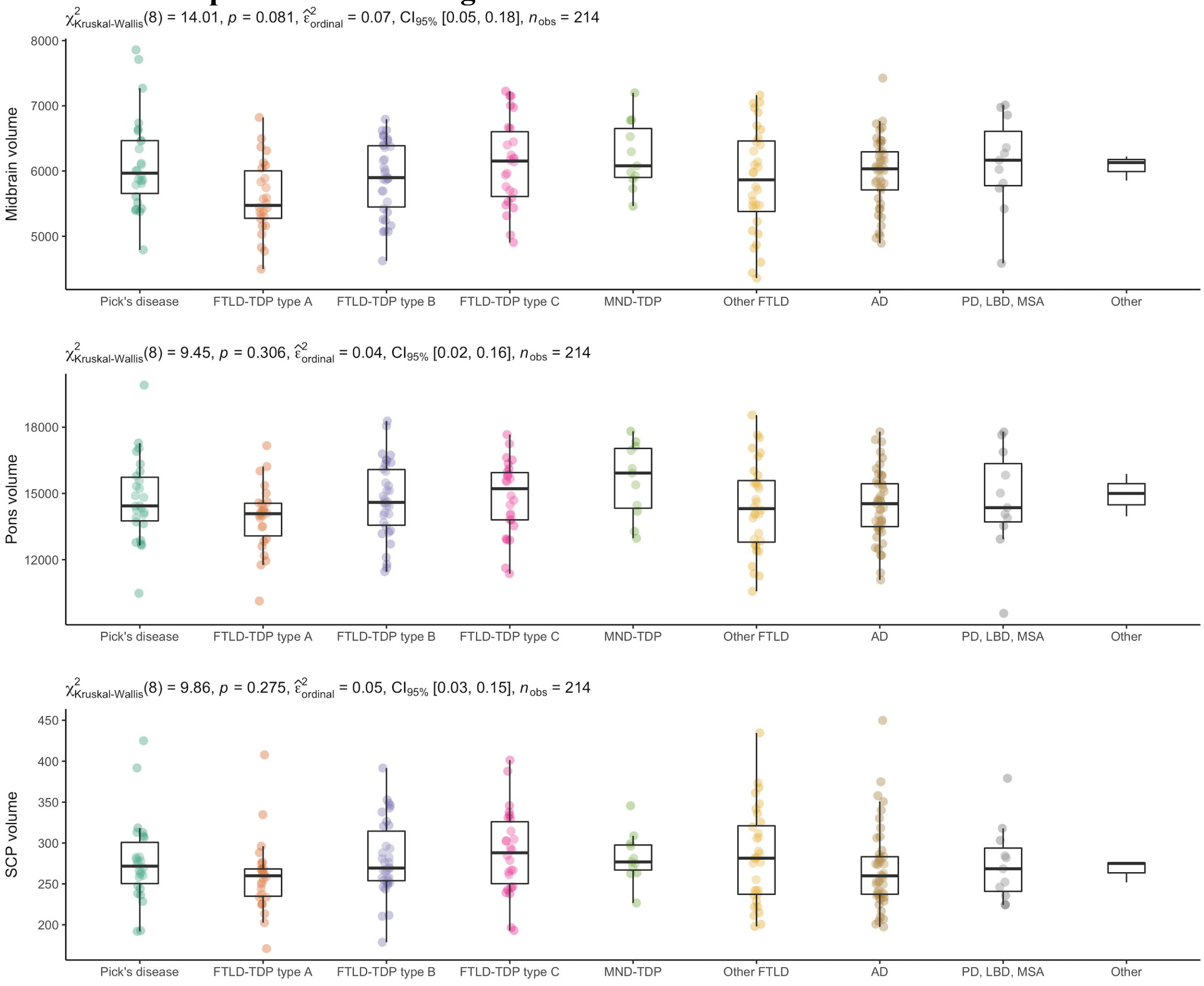

Horizontal lines indicate medians; boxes, quartile 1 to quartile 3; whiskers, minimum to maximum values; and dots, individual participant values.

Abbreviations: FTLD=frontotemporal lobar degeneration; TDP=TAR DNA binding protein 43; MND=motor neuron disease; AD=Alzheimer disease; PD=Parkinson disease; LBD=Lewy Body dementia; MSA=multiple system atrophy

© 2022 Illán-Gala I et al. *JAMA Network Open.*

eFigure 8. Group Comparison of Brainstem Measures of Atrophy in the Subgroup of Participants With PSP-RS or Probable CBS at Diagnosis

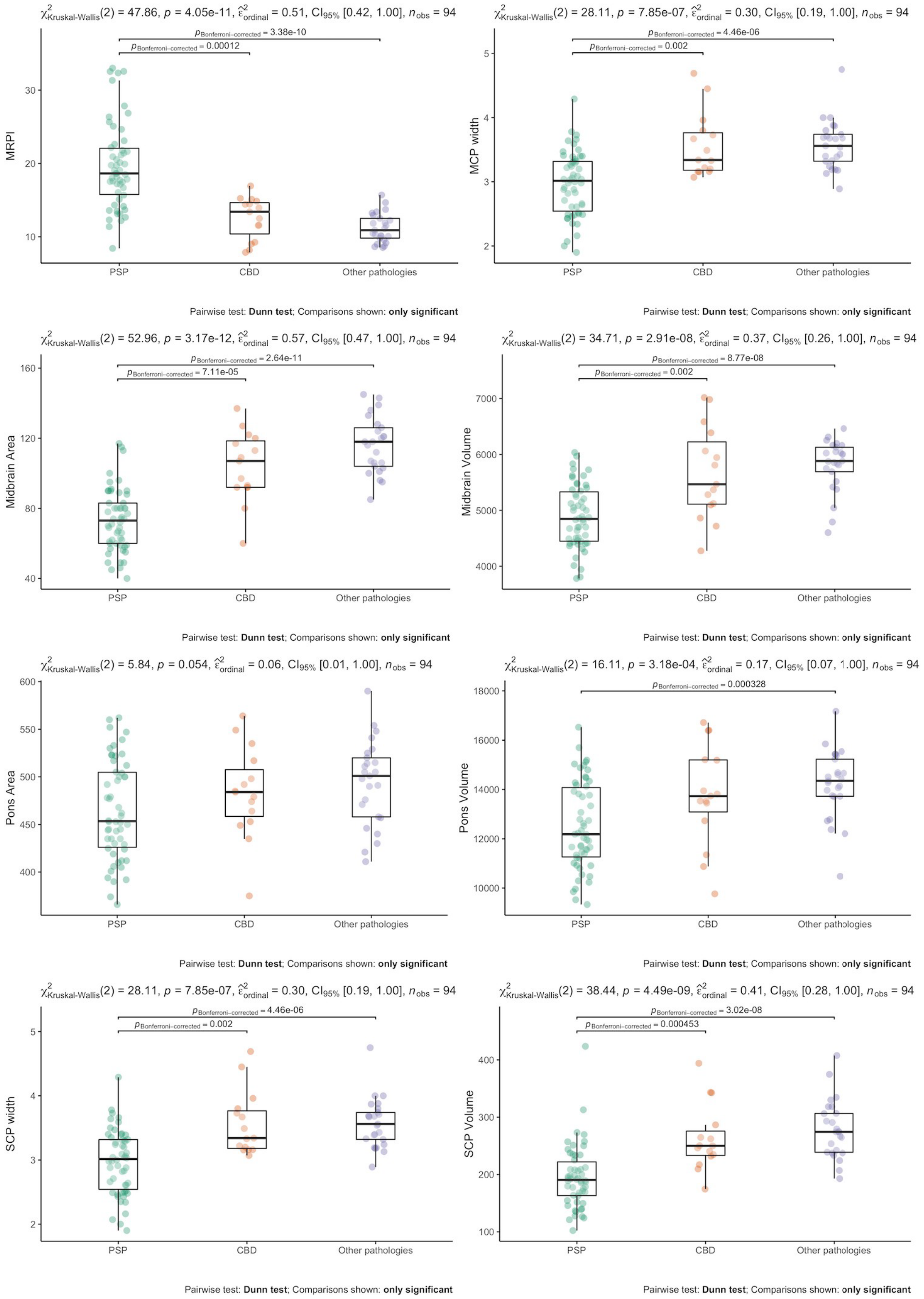

Horizontal lines indicate medians; boxes, quartile 1 to quartile 3; whiskers, minimum to maximum values; and dots, individual participant values.

Abbreviations: CBD=corticobasal disease; PSP=progressive supranuclear palsy; ns=non-significant; MCP=middle cerebellar peduncle; MRPI=magnetic resonance parkinsonism index; SCP=superior cerebellar peduncle

© 2022 Illán-Gala I et al. *JAMA Network Open.*

eFigure 9. Group Comparison of Brainstem Measures of Atrophy in the Subgroup of Participants Without PSP-RS or Probable CBS at Diagnosis

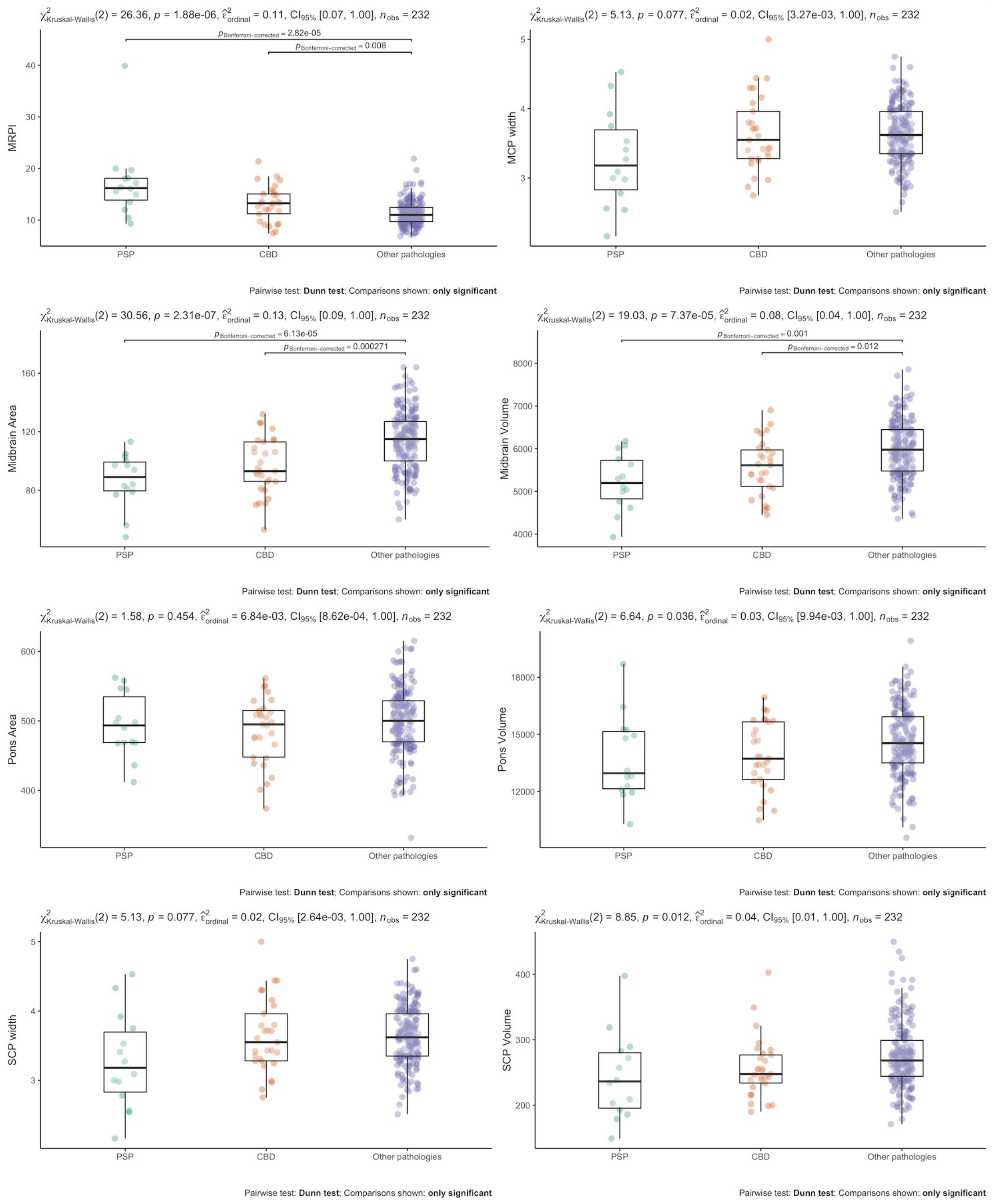

Horizontal lines indicate medians; boxes, quartile 1 to quartile 3; whiskers, minimum to maximum values; and dots, individual participant values.

Abbreviations: CBD=corticobasal disease; PSP=progressive supranuclear palsy; ns=non-significant; MCP=middle cerebellar peduncle; MRPI=magnetic resonance parkinsonism index; SCP=superior cerebellar peduncle.

© 2022 Illán-Gala I et al. *JAMA Network Open.*

eFigure 10. Multinomial Logistic Regression Model Considering Adjusted MRPI-Derived Measures of Brainstem Atrophy and Other Measures of Cortical and Subcortical Atrophy

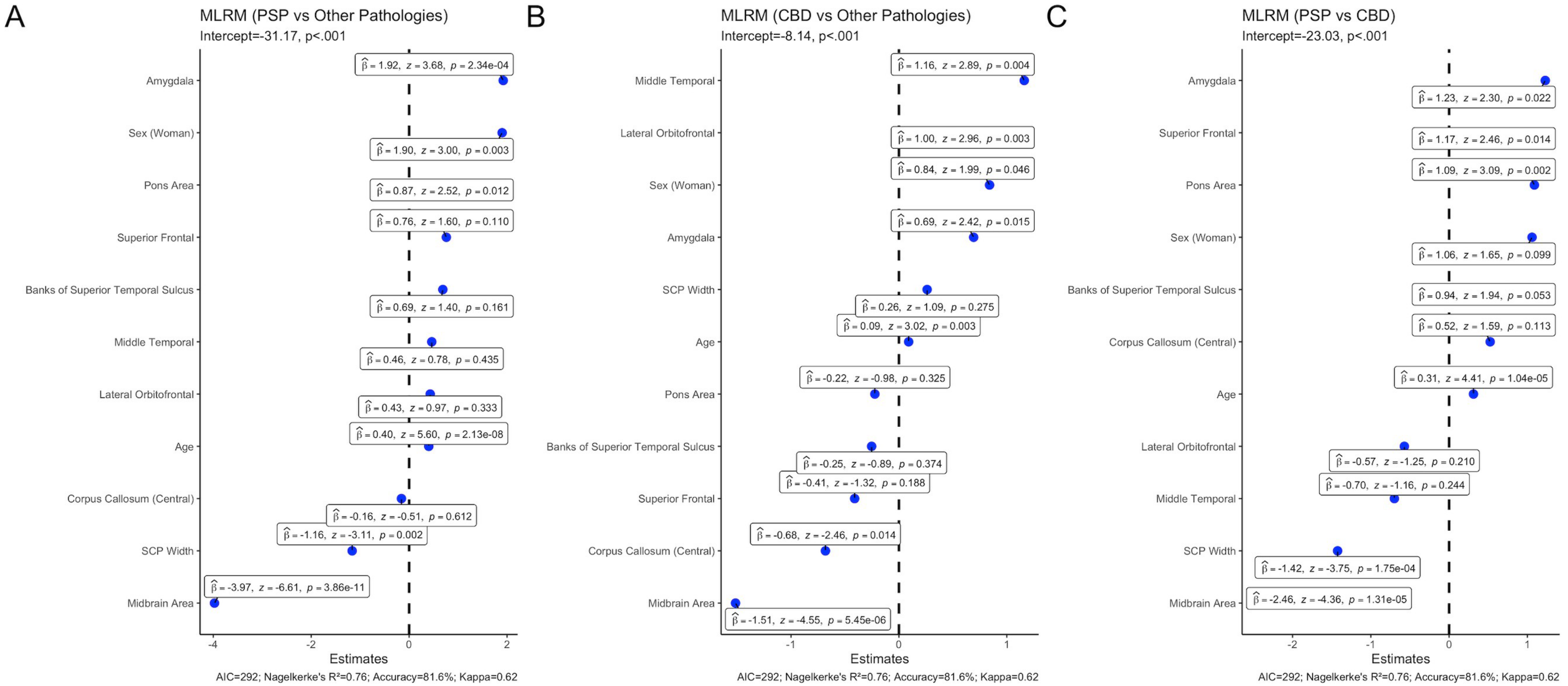

Multinomial logistic regression model considering age-, sex-, TIV- and scanner-adjusted MRPI-derived measures of brainstem atrophy and other cortical and subcortical measures for the discrimination between: **A)** PSP and Other Pathologies (without CBD); **B)** CBD and other pathologies (without PSP), and **C)** PSP and CBD. The effect of age, sex, TIV, and MRI scan was regressed out from all MRI-derived measures included in this model. Neuroimaging measures were also standardized to facilitate the comparison of their relative weight in the model. Five-fold cross-validation showed a global accuracy of 81.6% (Kappa=0.62).

Abbreviations: 4RT= four-repeat tau isoform tauopathies; AIC=Akaike information criterion; CBD=corticobasal disease; MLRM=multinomial logistic regression model; MRPI=magnetic resonance parkinsonism index; PPV=positive predictive value; PSP=progressive supranuclear palsy; SCP=superior cerebellar peduncle; TIV=total intracranial volume.

© 2022 Illán-Gala I et al. *JAMA Network Open.*

eFigure 11. Multinomial Logistic Regression Model Considering Adjusted Freesurfer-Derived Measures of Brainstem Atrophy and Other Measures of Cortical and Subcortical Atrophy

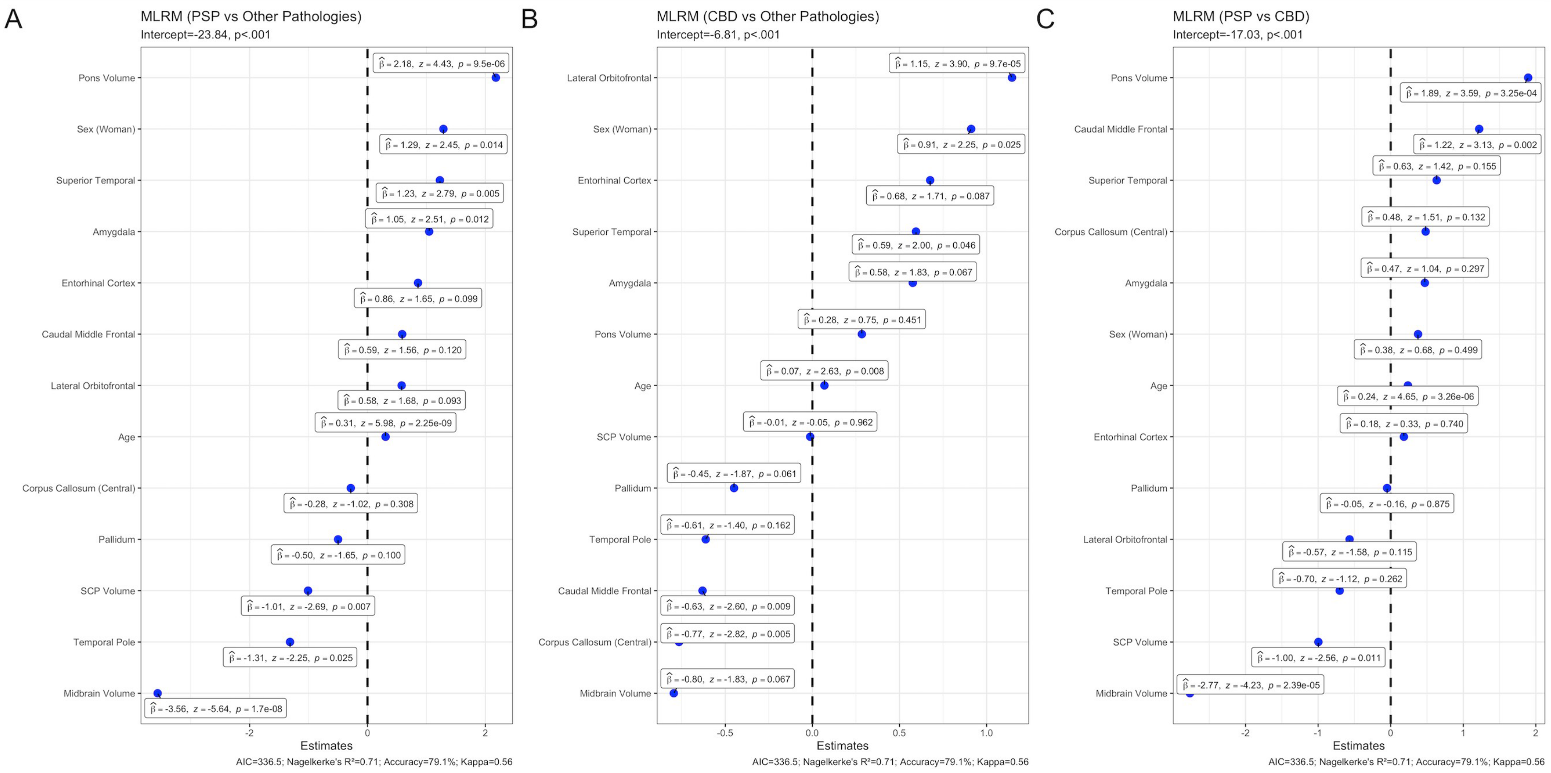

Multinomial logistic regression model considering age-, sex-, TIV- and scanner-adjusted Freesurfer-derived measures of brainstem atrophy and other measures of cortical and subcortical atrophy for the discrimination between: **A)** PSP and Other Pathologies (without CBD); **B)** CBD and other pathologies (without PSP), and **C)** PSP and CBD. The effect of age, sex, TIV, and MRI scan was regressed out from all MRI-derived measures included in this model. Neuroimaging measures were also standardized to facilitate the comparison of their relative weight in the model. Five-fold cross-validation showed an accuracy of 79.1% (Kappa=0.56).

Abbreviations: 4RT= four-repeat tau isoform tauopathies; AIC=Akaike information criterion; CBD=corticobasal disease; MLRM=multinomial logistic regression model; MRPI=magnetic resonance parkinsonism index; PPV=positive predictive value; PSP=progressive supranuclear palsy; SCP=superior cerebellar peduncle; TIV=total intracranial volume.

© 2022 Illán-Gala I et al. *JAMA Network Open.*

eFigure 12. Multinomial Logistic Regression Models Considering Raw MRPI-Derived Measures of Brainstem Atrophy and Other Measures of Cortical and Subcortical Atrophy

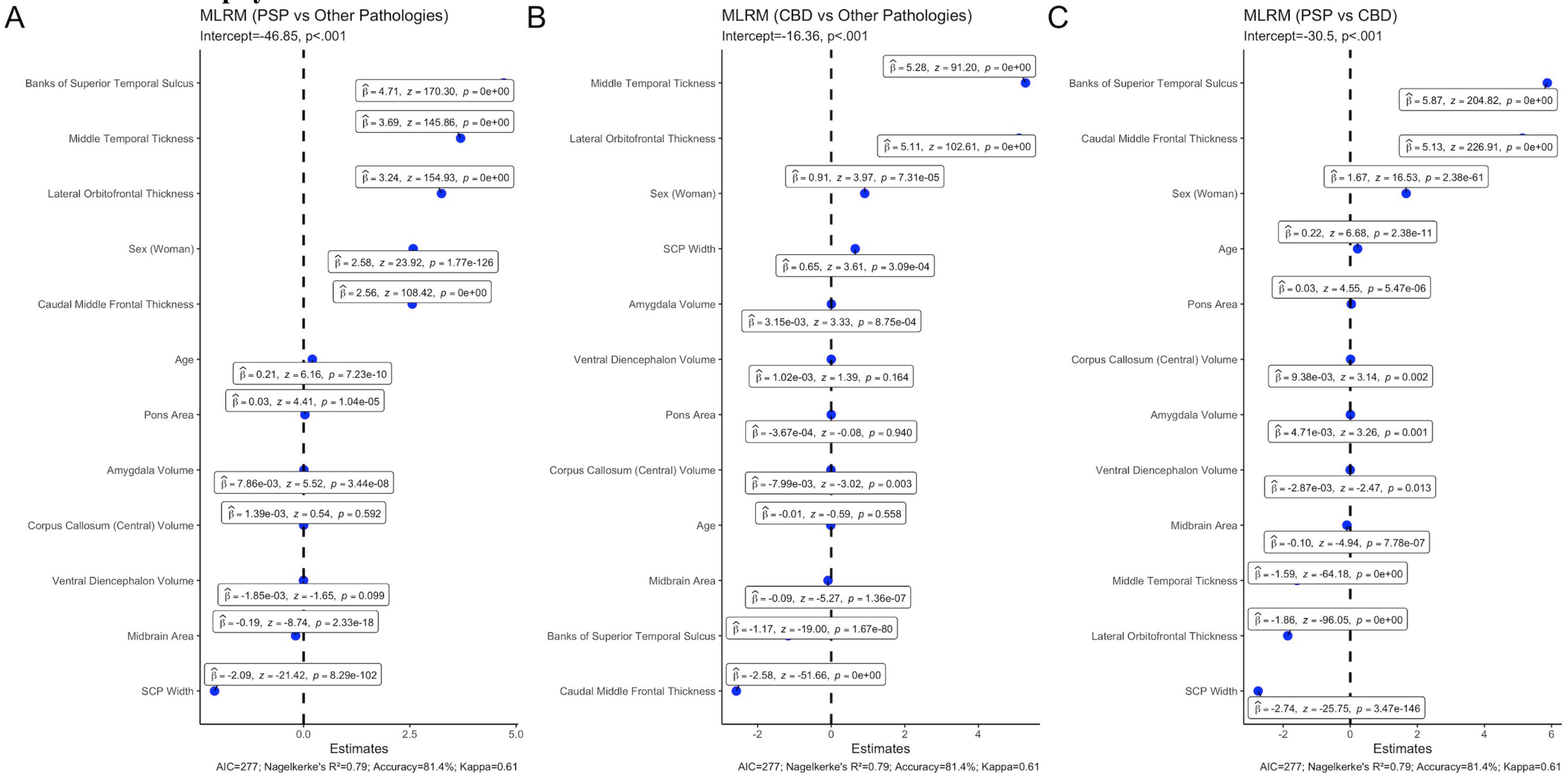

Multinomial logistic regression model considering raw MRPI-derived measures of brainstem atrophy and other cortical and subcortical measures for the discrimination between **A)** PSP and Other Pathologies (without CBD); **B)** CBD and other pathologies (without PSP), and **C)** PSP and CBD. In this model, we included raw neuroimaging measurements and MRI-derived measurements were not normalized to allow the calculation of predicted probabilities in other cohorts. Five-fold cross-validation of this model showed a global accuracy of 81.4% (Kappa=0.61).
Abbreviations: 4RT= four-repeat tau isoform tauopathies; AIC=Akaike information criterion; CBD=corticobasal disease; MLRM=multinomial logistic regression model; MRPI=magnetic resonance parkinsonism index; PPV=positive predictive value; PSP=progressive supranuclear palsy; SCP=superior cerebellar peduncle; TIV=total intracranial volume.

© 2022 Illán-Gala I et al. *JAMA Network Open.*

eFigure 13. Multinomial Logistic Regression Model Considering Raw Freesurfer-Derived Measures of Brainstem Atrophy and Other Measures of Cortical and Subcortical Atrophy

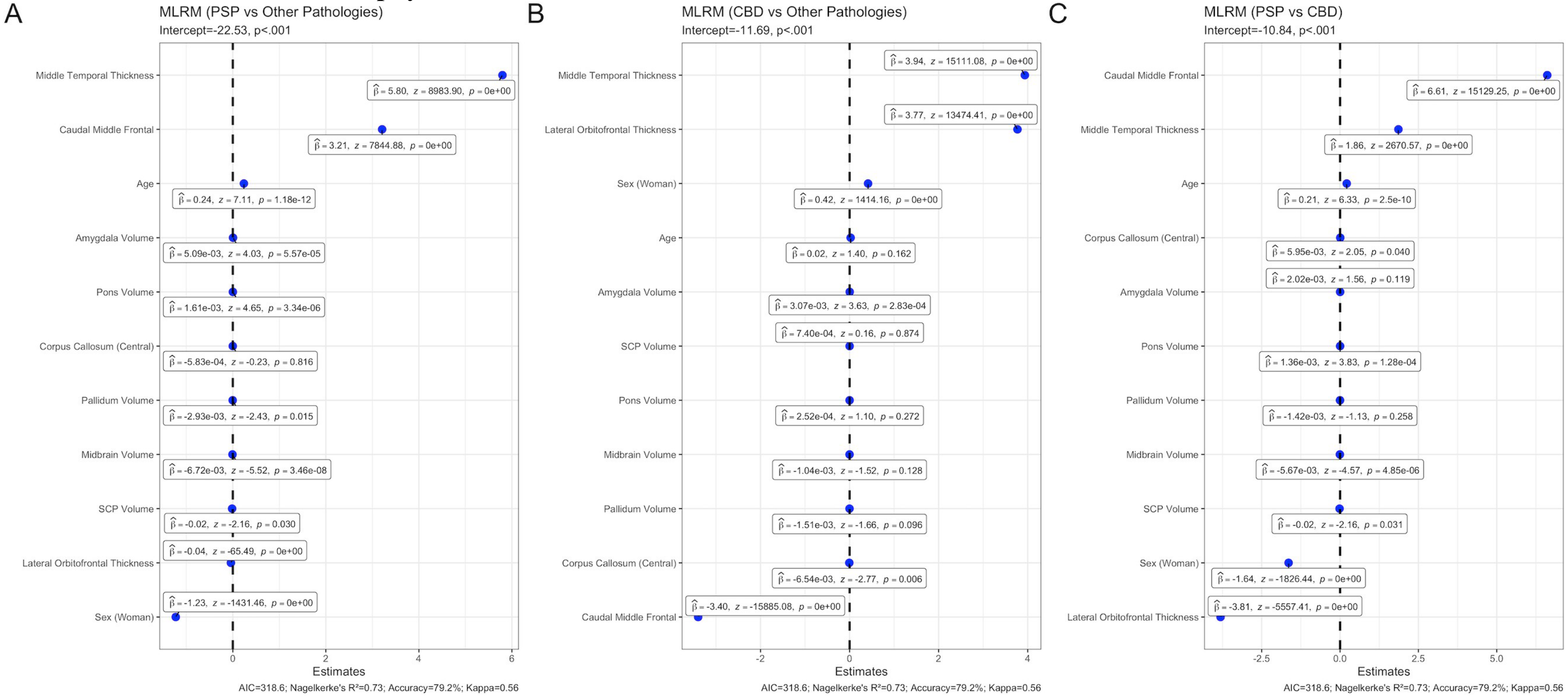

Multinomial logistic regression model considering raw Freesurfer-derived measures of brainstem atrophy and other cortical and subcortical measures for the discrimination between **A)** PSP and Other Pathologies (without CBD); **B)** CBD and other pathologies (without PSP), and **C)** PSP and CBD. In this model, we included raw neuroimaging measurements and MRI-derived measurements were not normalized to allow the calculation of predicted probabilities in other cohorts. Five-fold cross-validation of this model showed a global accuracy of 79.2% (Kappa=0.56).

Abbreviations: 4RT= four-repeat tau isoform tauopathies; AIC=Akaike information criterion; CBD=corticobasal disease; MLRM=multinomial logistic regression model; MRPI=magnetic resonance parkinsonism index; PPV=positive predictive value; PSP=progressive supranuclear palsy; SCP=superior cerebellar peduncle; TIV=total intracranial volume.

© 2022 Illán-Gala I et al. *JAMA Network Open.*

eFigure 14. ROC Analyses of Relevant Measures of Cerebral Atrophy in the Subgroups of Participants With and Without a Diagnosis of PSP-RS or Probable CBS

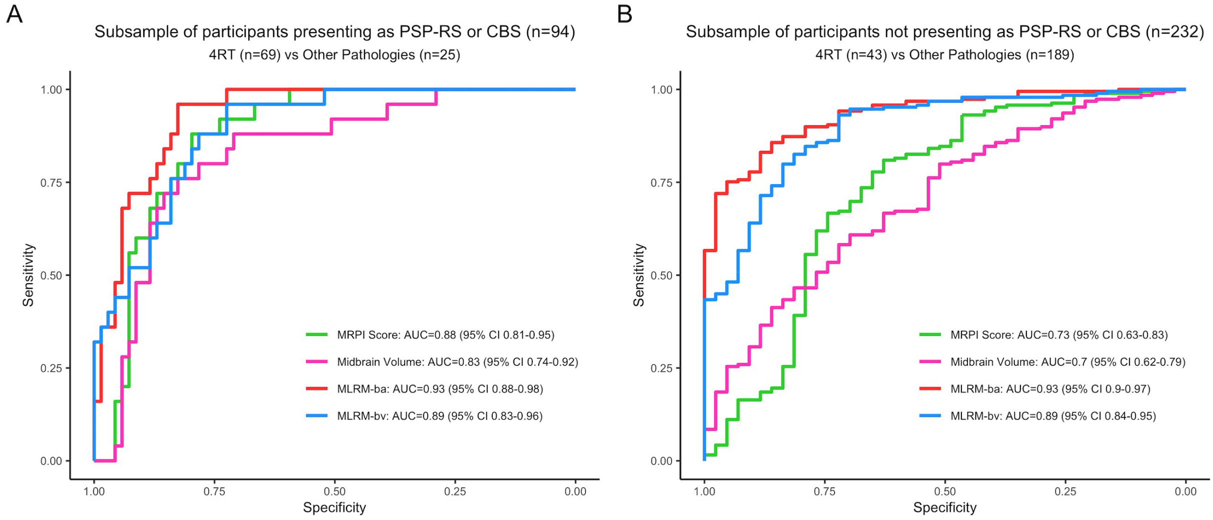

ROC curves of key measures of brainstem atrophy (MRPI score and the midbrain volume) and MLRM combining cortical and subcortical measures to discriminate between 4RT and other participants in **A)** the subgroup of participants presenting as PSP-RS or CBS (n=94) at MRI and **B)** the rest of participants without PSP-RS or CBS at MRI (n=231).

Abbreviations: 4RT= four-repeat tau isoform tauopathies; MLRM=multinomial logistic regression model; MLRM-ba=multinomial logistic regression model including brainstem areas; MLRM-bv=multinomial logistic regression model including brainstem volumes; CBS=corticobasal syndrome; MRPI=magnetic resonance parkinsonism index; PSP-RS=Richardson's syndrome.

© 2022 Illán-Gala I et al. *JAMA Network Open.*

eTable 1. Predominant Phenotype at MRI for Each Autopsy Group

Predominant phenotype at MRI	PSP (N=68)	CBD (N=44)	Other pathologies (N=214)
AD	2 (2.9%)	4 (9.1%)	12 (5.6%)
bvFTD	2 (2.9%)[a]	10 (22.7%)[b]	97 (45.3%)[c]
CBS	11 (16.2%)	12 (27.3%)[d]	22 (10.3%)[e]
LBD	0 (0%)	0 (0%)	10 (4.7%)
lvPPA	0 (0%)[d]	0 (0%)	21 (9.8%)[f]
MCI	5 (7.4%)[d]	5 (11.4%)[d]	0 (0%)[c]
MND	0 (0%)	0 (0%)	11 (5.1%)
nfvPPA	5 (7.4%)	9 (20.5%)[d]	11 (5.1%)[e]
PPA	0 (0%)	1 (2.3%)	4 (1.9%)
PSP-RS	43 (63.2%)[a]	3 (6.8%)[f]	3 (1.4%)[f]
svPPA	0 (0%)[d]	0 (0%)	23 (10.7%)[f]
**Last diagnosis (participants with follow-up)**	**PSP (N=56)**	**CBD (N=35)**	**Other pathologies (N=153)**
AD	0 (0%)	1 (2.9%)	24 (16%)
bvFTD	1 (1.8%)[a]	8 (23%)[b]	60 (39%)[c]
CBS	6 (11%)	14 (40%)[d]	13 (8.5%)[e]
LBD	0 (0%)	0 (0%)	8 (5.2%)
lvPPA	0 (0%)	0 (0%)	7 (4.6%)
Vascular	0 (0%)	0 (0%)	1 (0.7%)
MND	0 (0%)	0 (0%)	4 (2.6%)
nfvPPA	4 (7.1%)	8 (23%)	11 (7.2%)
PSP-RS	45 (80%)	4 (11%)	2 (1.3%)
svPPA	0 (0%)	0 (0%)	23 (15%)

The PPA group included participants with prominent language features non-fitting any PPA variant. The Lewy Body Disease (LBD) group included participants that received a diagnosis of Parkinson's disease (n=2), Multiple System Atrophy (n=2) and Lewy body Dementia (n=6).
a: Different from CBD and other pathologies (P<0.05, Bonferroni corrected)
b: Different from PSP and other pathologies (P<0.05, Bonferroni corrected)
c: Different from PSP and CBD (P<0.05, Bonferroni corrected)
d: Different from other pathologies (P<0.05, Bonferroni corrected)
e: Different from CBD (P<0.05, Bonferroni corrected)
f: Different from PSP (P<0.05, Bonferroni corrected)
Abbreviations: AD=Alzheimer's disease; bvFTD=behavioral variant of frontotemporal dementia; CBS=corticobasal syndrome; LBD=Lewy body disease; lvPPA=logopenic variant of primary progressive aphasia; MCI=mild cognitive impairment; MDD=motor neuron disease; nfvPPA=non-fluent/agrammatic variant of primary progressive aphasia; PPA=primary progressive aphasia; PSP-RS=Richardson's syndrome; svPPA=semantic variant of primary progressive aphasia.

© 2022 Illán-Gala I et al. *JAMA Network Open.*

eTable 2. Comparison of Measures of Brainstem Atrophy Across Groups

Measure	Group				Effect Size (Cohen's d)			
	PSP n=68	CBD n=44	4RT n=112	Other n=214	PSP vs Other	CBD vs Other	4RT vs Other	PSP vs CBD
Pons Area, mm^2	471 (51.6)	485 (47.2)	476 (50.1)	499 (46.9)	-0.3	NS	-0.3	NS
Midbrain Area, mm^2	76.1 (18.6)	99.0 (19.7)	85.1 (22.1)	114 (19.6)	-1.5	-0.6	-1.1	-0.8
MCP Width, mm	8.63 (0.939)	9.09 (1.03)	8.81 (0.997)	9.06 (0.870)	NS	NS	NS	NS
SCP Width, mm	3.03 (0.554)	3.61 (0.513)	3.26 (0.607)	3.63 (0.405)	-1.1	NS	-0.6	-1
MRPI Score	19.2 (6.19)	13.0 (3.21)	16.7 (6.03)	11.3 (2.29)	1.5	0.5	1.1	1.1
Medulla Volume, mm^3	3990 (453)	4440 (493)	4170 (517)	4450 (591)	-0.6	NS	-0.4	-0.8
Pons Volume, mm^3	12800 (1920)	13800 (1840)	13200 (1950)	14600 (1760)	-0.7	-0.4	-0.6	-0.4
SCP Volume, mm^3	205 (59.8)	259 (49.5)	226 (61.6)	276 (49.0)	-1.1	-0.3	-0.8	-0.8
Midbrain, mm^3	4960 (594)	5600 (705)	5210 (710)	5950 (648)	-1.4	-0.5	-1	-0.8
Whole brainstem, mm^3	21900 (2810)	24100 (2930)	22800 (3040)	25300 (2780)	-0.9	-0.4	-0.7	-0.6

Effects sizes (Cohen's d values) are shown for different group comparisons after accounting for age, sex, TIV and MRI scan. Only statistically significant effect sizes are shown (Bonferroni, $P<0.05$).

Abbreviations: 4RT= four-repeat tau isoform tauopathies; CBD=corticobasal disease; MCP=middle cerebellar peduncle; PSP=progressive supranuclear palsy; NS=non-significant; MRPI=magnetic resonance parkinsonism index; SCP=superior cerebellar peduncle.

© 2022 Illán-Gala I et al. *JAMA Network Open.*

eTable 3. Group Comparison of Neuroimaging Measures

Variables[a]	PSP (N=68)	CBD (N=44)	4RT (N=112)	Other pathologies (N=214)	PSP vs CBD vs Other pathologies	CBD vs Other	PSP vs CBD	PSP vs Other	4RT vs Other
Pons Area (mm2)	471 (51.6)	485 (47.2)	476 (50.1)	499 (46.9)	0.002	0.43	0.43	0.006	<0.001
Midbrain Area (mm2)	76.1 (18.6)	99.0 (19.7)	85.1 (22.1)	114 (19.6)	<0.001	<0.001	<0.001	<0.001	<0.001
MCP Width (mm)	8.63 (0.939)	9.09 (1.03)	8.81 (0.997)	9.06 (0.870)	0.005	1	0.4	0.009	0.011
SCP Width (mm)	3.03 (0.554)	3.61 (0.513)	3.26 (0.607)	3.63 (0.405)	<0.001	1	<0.001	<0.001	<0.001
MRPI Score	19.2 (6.19)	13.0 (3.21)	16.7 (6.03)	11.3 (2.29)	<0.001	0.007	<0.001	<0.001	<0.001
Medulla Volume (mm3)	3990 (453)	4440 (493)	4170 (517)	4450 (591)	<0.001	1	<0.001	<0.001	<0.001
Pons Volume (mm3)	12800 (1920)	13800 (1840)	13200 (1950)	14600 (1760)	<0.001	0.2	0.018	<0.001	<0.001
SCP Volume (mm3)	205 (59.8)	259 (49.5)	226 (61.6)	276 (49.0)	<0.001	0.025	<0.001	<0.001	<0.001
Midbrain Volume (mm3)	4960 (594)	5600 (705)	5210 (710)	5950 (648)	<0.001	0.03	<0.001	<0.001	<0.001
Whole brainstem volume (mm3)	21900 (2810	24100 (2930)	22800 (3040)	25300 (2780)	<0.001	0.239	0.002	<0.001	<0.001
Banks of Superior Temporal Sulcus thickness (mm)	2.41 (0.127)	2.35 (0.157)	2.39 (0.142)	2.27 (0.188)	<0.001	0.015	0.398	<0.001	<0.001
Caudal Anterior Cingulate thickness (mm)	2.29 (0.202)	2.24 (0.238)	2.27 (0.217)	2.25 (0.203)	0.114	1	1	0.328	0.087
Caudal Middle Frontal thickness (mm)	2.31 (0.164)	2.18 (0.200)	2.26 (0.189)	2.21 (0.207)	<0.001	1	0.017	0.001	0.012
Cuneus thickness (mm)	1.86 (0.0986)	1.89 (0.106)	1.87 (0.101)	1.87 (0.128)	0.655	1	1	1	0.741
Entorhinal thickness (mm)	3.10 (0.285)	3.03 (0.322)	3.07 (0.300)	2.67 (0.486)	<0.001	<0.001	0.45	<0.001	<0.001
Fusiform thickness (mm)	2.60 (0.125)	2.61 (0.178)	2.60 (0.148)	2.47 (0.208)	<0.001	<0.001	0.604	<0.001	<0.001
Inferior Parietal thickness (mm)	2.30 (0.110)	2.27 (0.132)	2.29 (0.119)	2.21 (0.181)	0.005	0.293	1	0.013	0.001
Inferior Temporal thickness (mm)	2.64 (0.128)	2.66 (0.154)	2.65 (0.138)	2.46 (0.202)	<0.001	<0.001	1	<0.001	<0.001
Isthmus thickness (mm)	2.14 (0.137)	2.17 (0.163)	2.16 (0.148)	2.08 (0.152)	0.003	0.035	0.971	0.115	<0.001
Lateral Occipital thickness (mm)	2.14 (0.0980)	2.18 (0.134)	2.16 (0.114)	2.11 (0.151)	0.003	0.01	0.299	0.299	0.002
Lateral Orbitofrontal thickness (mm)	2.50 (0.147)	2.48 (0.158)	2.49 (0.151)	2.37 (0.186)	<0.001	0.003	1	<0.001	<0.001
Lingual thickness (mm)	1.96 (0.0908)	1.99 (0.123)	1.97 (0.105)	1.95 (0.126)	0.065	0.188	0.452	1	0.108
Medial Orbitofrontal thickness (mm)	2.32 (0.132)	2.32 (0.171)	2.32 (0.148)	2.22 (0.206)	<0.001	0.021	1	<0.001	<0.001
Middle Temporal thickness (mm)	2.65 (0.119)	2.63 (0.166)	2.64 (0.139)	2.45 (0.216)	<0.001	<0.001	1	<0.001	<0.001
Parahippocampal thickness (mm)	2.58 (0.221)	2.59 (0.221)	2.58 (0.220)	2.40 (0.282)	<0.001	<0.001	1	<0.001	<0.001
Paracentral thickness (mm)	2.24 (0.171)	2.23 (0.171)	2.24 (0.170)	2.18 (0.182)	0.024	0.582	1	0.102	0.005
Pars Opercularis thickness (mm)	2.38 (0.149)	2.30 (0.196)	2.35 (0.172)	2.27 (0.196)	<0.001	0.498	0.15	<0.001	<0.001
Pars Orbitalis thickness (mm)	2.56 (0.185)	2.53 (0.231)	2.55 (0.204)	2.41 (0.233)	<0.001	0.033	1	<0.001	<0.001
Pars Triangularis thickness (mm)	2.27 (0.124)	2.24 (0.172)	2.26 (0.145)	2.19 (0.177)	0.001	0.179	1	0.003	<0.001
Pericalcarine thickness (mm)	1.61 (0.123)	1.65 (0.122)	1.63 (0.124)	1.63 (0.141)	0.417	1	1	1	0.914
Postcentral thickness (mm)	2.00 (0.103)	1.99 (0.144)	1.99 (0.120)	1.98 (0.155)	0.371	1	1	1	0.316
Posterior Cingulate thickness (mm)	2.28 (0.131)	2.24 (0.123)	2.26 (0.129)	2.19 (0.146)	<0.001	0.169	0.161	<0.001	<0.001
Precentral thickness (mm)	2.24 (0.182)	2.20 (0.153)	2.23 (0.172)	2.19 (0.206)	0.46	1	1	1	0.445
Precuneus thickness (mm)	2.25 (0.121)	2.22 (0.139)	2.24 (0.129)	2.19 (0.168)	0.113	1	1	0.336	0.093
Rostral Anterior Cingulate thickness (mm)	2.63 (0.212)	2.62 (0.226)	2.62 (0.217)	2.50 (0.247)	<0.001	0.004	1	0.001	<0.001
Rostral Middle Frontal thickness (mm)	2.26 (0.137)	2.21 (0.138)	2.24 (0.139)	2.16 (0.173)	<0.001	0.128	0.128	<0.001	<0.001
Superior Frontal thickness (mm)	2.45 (0.170)	2.37 (0.183)	2.42 (0.179)	2.35 (0.198)	<0.001	0.944	0.125	<0.001	<0.001
Superior Parietal thickness (mm)	2.13 (0.127)	2.06 (0.131)	2.10 (0.132)	2.06 (0.172)	0.018	1	0.065	0.143	0.259
Superior Temporal thickness (mm)	2.58 (0.134)	2.58 (0.176)	2.58 (0.151)	2.43 (0.202)	<0.001	0.002	1	<0.001	<0.001
Supramarginal thickness (mm)	2.35 (0.110)	2.31 (0.132)	2.33 (0.120)	2.27 (0.185)	0.005	0.536	0.536	0.009	0.002

© 2022 Illán-Gala I et al. *JAMA Network Open.*

Frontal Pole thickness (mm)	2.62 (0.224)	2.63 (0.236)	2.63 (0.228)	2.54 (0.272)	0.009	0.078	1	0.178	0.003
Temporal Pole thickness (mm)	3.41 (0.315)	3.36 (0.299)	3.39 (0.309)	3.03 (0.499)	<0.001	<0.001	0.975	<0.001	<0.001
Transverse Temporal thickness (mm)	2.27 (0.206)	2.31 (0.204)	2.28 (0.206)	2.26 (0.221)	0.228	0.792	1	1	0.227
Insula thickness (mm)	2.83 (0.142)	2.82 (0.176)	2.82 (0.156)	2.66 (0.203)	<0.001	<0.001	1	<0.001	<0.001
Mean cortical thickness	2.32 (0.0962)	2.30 (0.109)	2.31 (0.102)	2.23 (0.122)	<0.001	0.004	1	<0.001	<0.001
Lateral Ventricle Volume (mm3)	20000 (8720)	22200 (7430)	20900 (8280)	23400 (11400)	0.109	1	0.587	0.431	0.235
Inferior Lateral Ventricle Volume (mm3)	875 (519)	1170 (1060)	990 (787)	1430 (1140)	<0.001	0.028	0.76	<0.001	<0.001
Cerebellum White Matter Volume (mm3)	12100 (1820)	13600 (2160)	12700 (2090)	13900 (2320)	<0.001	1	0.001	<0.001	<0.001
Cerebellum Cortex Volume (mm3)	49700 (5760)	51900 (5630)	50600 (5780)	51800 (7010)	0.068	1	1	0.177	0.041
Thalamus Volume (mm3)	5890 (624)	6280 (887)	6040 (760)	6650 (994)	<0.001	0.115	0.115	<0.001	<0.001
Caudate Volume (mm3)	3140 (565)	2950 (615)	3060 (590)	2980 (662)	0.153	1	0.815	0.535	0.175
Putamen Volume (mm3)	4010 (538)	3890 (657)	3960 (587)	3850 (780)	0.285	1	1	1	0.434
Pallidum Volume (mm3)	1560 (226)	1640 (291)	1600 (255)	1820 (288)	<0.001	0.003	1	<0.001	<0.001
Hippocampus Volume (mm3)	3710 (392)	3620 (499)	3670 (437)	3370 (568)	<0.001	0.071	0.835	<0.001	<0.001
Amygdala Volume (mm3)	1480 (207)	1410 (209)	1450 (209)	1270 (299)	<0.001	0.002	0.234	<0.001	<0.001
Accumbens Area Volume (mm3)	376 (78.0)	356 (91.5)	368 (83.8)	325 (104)	<0.001	0.139	0.871	<0.001	<0.001
Ventral Diencephalon Volume (mm3)	3180 (387)	3510 (460)	3310 (445)	3680 (489)	<0.001	0.301	0.013	<0.001	<0.001
Vessels Volume (mm3)	33.8 (20.1)	36.1 (21.0)	34.7 (20.4)	35.2 (34.8)	0.545	1	1	1	0.322
Choroid Plexus Volume (mm3)	856 (223)	954 (228)	894 (229)	919 (235)	0.137	1	0.453	0.453	0.333
Third Ventricle Volume (mm3)	2170 (682)	2230 (644)	2190 (665)	2080 (653)	0.205	1	1	1	0.072
Fourth Ventricle Volume (mm3)	2300 (673)	2160 (652)	2250 (665)	2030 (654)	0.004	0.826	0.939	0.012	0.002
Fifth Ventricle Volume (mm3)	0.0632 (0.521)	0 (0)	0.0384 (0.406)	0.0780 (0.563)	0.119	1	1	0.388	0.053
WM Hyperintensities Volume (mm3)	4070 (3750)	2990 (1650)	3650 (3130)	3740 (3830)	0.498	1	1	1	0.574
Corpus Callosum (Posterior) Volume (mm3)	986 (226)	938 (172)	967 (207)	978 (208)	0.529	1	1	1	0.375
Corpus Callosum (Mid Posterior) Volume (mm3)	484 (153)	480 (115)	482 (139)	505 (128)	0.154	1	1	0.617	0.058
Corpus Callosum (Central) Volume (mm3)	412 (112)	382 (64.4)	400 (97.0)	443 (112)	<0.001	<0.001	0.536	0.075	<0.001
Corpus Callosum (Mid Anterior) Volume (mm3)	423 (98.2)	375 (101)	404 (102)	426 (120)	0.059	0.216	0.216	1	0.288
Corpus Callosum (Anterior) Volume (mm3)	854 (140)	820 (201)	841 (167)	849 (199)	0.808	1	1	1	0.845

Bonferroni-corrected *P*-values are shown for robust t-test (4RT vs Other pathologies), robust ANOVA (PSP vs CBD vs Other pathologies) and post-hoc group comparison (Yuen's trimmed means).

a: Results are expressed as mean (SD).

Abbreviations: 4RT= four-repeat tau isoform tauopathies; CBD=corticobasal disease; PSP=progressive supranuclear palsy; MCP=middle cerebellar peduncle; MRPI=magnetic resonance parkinsonism index; SCP=superior cerebellar peduncle; WM=white matter;

© 2022 Illán-Gala I et al. *JAMA Network Open.*
